# The Effectiveness of Curcumin, Resveratrol, and Silymarin on MASLD: A Systematic Review and Meta‐Analysis

**DOI:** 10.1002/fsn3.4595

**Published:** 2024-11-14

**Authors:** Qian Huang, Ziming An, Xin Xin, Xiaojun Gou, Xiaoting Tian, Yiyang Hu, Zubing Mei, Qin Feng

**Affiliations:** ^1^ Institute of Liver Diseases Shuguang Hospital Affiliated to Shanghai University of Traditional Chinese Medicine Shanghai China; ^2^ School of Basic Medicine Shaanxi University of Chinese Medicine Xianyang Shaanxi China; ^3^ Central Laboratory Baoshan District Hospital of Integrated Traditional Chinese and Western Medicine of Shanghai Shanghai China; ^4^ Shanghai Institute of Materia Medica, Chinese Academy of Sciences Shanghai China; ^5^ Institute of Anorectal Diseases Shuguang Hospital Affiliated to Shanghai University of Traditional Chinese Medicine Shanghai China; ^6^ Shanghai Key Laboratory of Traditional Chinese Clinical Medicine Shanghai China; ^7^ Key Laboratory of Liver and Kidney Diseases Shanghai University of Traditional Chinese Medicine, Ministry of Education Shanghai China; ^8^ Central Laboratory Shuguang Hospital Affiliated to Shanghai University of Chinese Traditional Medicine Shanghai China

**Keywords:** curcumin, meta‐analysis, metabolic dysfunction‐associated steatotic liver disease, resveratrol, silymarin

## Abstract

Polyphenols, known for their potent antioxidant and anti‐inflammatory properties, have emerged as promising, natural, and safe complementary treatment options for metabolic‐associated steatotic liver disease (MASLD). Among these, curcumin, resveratrol, and silymarin are the most extensively studied; however, their differential effects on MASLD outcomes remain inconclusive. This systematic review and meta‐analysis of RCTs aimed to evaluate the efficacy of curcumin, resveratrol, and silymarin in patients with MASLD. A comprehensive search of seven databases was conducted up to September 2024. Odds ratios (OR), mean differences (MD), and standardized MD (SMD) with 95% confidence intervals (CI) were used to assess treatment effects. Primary outcomes included improvement in hepatic steatosis and ALT activity, while secondary outcomes included changes in AST activity, blood lipids, glucose, BMI, blood pressure, and TNF‐α. Twenty‐seven studies involving 1691 participants were included. Curcumin significantly improved hepatic steatosis compared to placebo (OR: 4.39, 95% CI: 1.45 to 13.27, *p* = 0.009), followed by resveratrol (OR: 3.18, 95% CI: 1.20 to 8.42, *p* = 0.02). Silymarin exhibited the strongest effect in reducing ALT levels (MD: −6.44 U/L, 95% CI: −10.03 to −2.85, *p* = 0.0004), with curcumin (MD: −5.88 U/L, 95% CI: −9.05 to −2.72, *p* = 0.0003) also showing significant reductions. A marked reduction in AST was observed with silymarin (MD: −6.99 U/L, 95% CI: −8.56 to −5.42, *p* < 0.00001), followed by curcumin (MD: −3.36 U/L, 95% CI: −5.35 to −1.36, *p* = 0.001). Furthermore, curcumin intake significantly improved metabolic indicators (TG, FBG, HOMA‐IR, and BMI). Resveratrol reduced FBG and DBP. Curcumin had the strongest effect on hepatic steatosis and improved both transaminase levels and metabolic markers. Silymarin demonstrated the greatest reduction in transaminase levels, while resveratrol showed modest benefits in steatosis and metabolic improvements. The three polyphenols appear as promising therapeutics for the treatment of MASLD.

## Graphical abstract

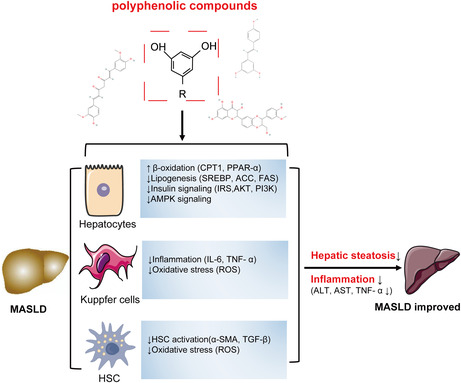

## 1 Introduction

With the global rise in type 2 diabetes mellitus (T2DM) and obesity, nonalcoholic fatty liver disease (NAFLD), now referred to as metabolic dysfunction‐associated steatotic liver disease (MASLD) (Rinella et al. 2023), has emerged as the leading cause of hepatocellular carcinoma (HCC) (Talamantes et al. 2023). MASLD represents a spectrum of disease that ranges from simple steatosis to metabolic dysfunction‐associated steatohepatitis (MASH), previously known as non‐alcoholic steatohepatitis (NASH), and may ultimately progress to fibrosis (Calzadilla Bertot and Adams 2016). Despite the increasing prevalence and severity of this condition, only one drug has been approved by the FDA for the treatment of NASH in the past 40 years, which is insufficient to address the substantial clinical need (Harrison et al. 2024). Consequently, there is an urgent need for the development of safe and effective therapeutic strategies to manage MASLD.

Polyphenols are a group of phytochemicals that share a common phenolic structure (Alberdi et al. 2013), such as curcumin and resveratrol, and have a long history of being used as functional foods, nutraceuticals, and pharmaceutical products. Enormous studies indicated that polyphenols are safe to be used in a variety of populations. A review summarized that they could alleviate oxidative stress, promote fatty acid beta‐oxidation, and modulate insulin resistance (Larussa et al. 2019; Williamson and Sheedy 2020). Furthermore, polyphenols can also protect liver (Khan and Mukhtar 2018). In animal studies, they improved liver steatogenesis, oxidative stress, and inflammation (Rodriguez‐Ramiro, Vauzour, and Minihane 2016; Van De Wier et al. 2017). Several clinical trials have evaluated several polyphenolic active ingredients that can affect MASLD (Jalali et al. 2020; Zeraattalab‐Motlagh, Jayedi, and Shab‐Bidar 2021).

In the current study, curcumin, resveratrol, and silymarin were the most investigated polyphenolic compounds for the treatment of MASLD. However, the studies were limited by relatively small sample sizes and inconsistent outcomes (Asghari et al. 2018; Panahi et al. 2017). Moreover, the efficacy of these three compounds has not been directly compared, making it difficult to draw definitive conclusions about their relative effectiveness.

Given the gaps in the current evidence, particularly the lack of comprehensive evaluation of curcumin, resveratrol, and silymarin in treating MASLD, a systematic review and meta‐analysis are warranted. Such an analysis would provide a more robust and evidence‐based understanding of their therapeutic potential in MASLD management.

## 2 Materials and Methods

We systematically identified relevant articles published before Sep. 2024 by searching PubMed, Cochrane Library, Embase, China Biology Medicine, Web of Science, China National Knowledge Internet, and Wanfang databases. Search terms included the keywords: (“NAFLD,” “non‐alcoholic fatty liver disease,” “non‐alcoholic steatohepatitis,” and “NASH”) and (“active ingredient,” “bioactive ingredients,” “active compounds,” “active components,” “natural compounds,” and “natural products”). To identify additional articles, we also manually looked for the references of reviews and relevant original studies.

This meta‐analysis was conducted using a random/fixed‐effect model, and the quality of articles was assessed using the Cochrane Risk of Bias 2.0. The manuscript was written according to the Preferred Reporting Items for Systematic Reviews and Meta‐Analyses (PRISMA) harm checklist. This study was registered in PROSPERO (CRD42022335681).

### 2.1 Selection Criteria

1. *Population*: individuals diagnosed with MASLD according to liver biopsy or noninvasive imaging modalities (Fibroscan or B‐ultrasound). Inclusion criteria included hepatic histology, B‐ultrasound, and Fibroscan. They are as follows: (1) Biopsy‐verified MASLD with a steatosis score ≥ 1, (2) B‐ultrasound indicated steatosis, and (3) controlled attenuation parameter (CAP) value > 263 dB/m (Petroff et al. 2021; Siddiqui et al. 2019).

The grades of hepatic steatosis are according to B‐ultrasound and biopsy: (i) based on B‐ultrasound: hepatic steatosis was graded as 0 (lack of fat accumulation), 1 (mild fat deposits), 2 (moderate fat deposits), and 3 (severe fat deposits) (Saadeh et al. 2002), (ii) based on liver biopsy: the distribution of steatosis for stages 0, 1, 2, and 3 was < 5%, 5%–33%, 33%–67%, and > 67%, respectively (Faghihzadeh et al. 2014).

All MASLD patients were ≥ 18 years old and had no primary systemic diseases or primary malignant tumors.

2 *Intervention*: Curcumin, resveratrol, and silymarin were administered at any form and dosage, with an intervention duration of ≥ 4 weeks.
3 *Comparison*: placebo.
4 *Outcomes*: (i) Primary outcomes were liver parameters: hepatic steatosis, alanine transaminase (ALT) activity. (ii) Secondary outcomes were aspartate aminotransferase (AST) activity, blood lipid indices [total cholesterol (TC), total glyceride (TG), high‐density lipoprotein cholesterol (HDL‐C), low‐density lipoprotein cholesterol (LDL‐C), blood glucose indicators [fasting plasma glucose (FPG), homeostasis model assessment‐insulin resistance (HOMA‐IR)], body mass index (BMI), blood pressure [systolic blood pressure (SBP), diastolic blood pressure (DBP)], and inflammatory factor [tumor necrosis factor‐α (TNF‐α)].

Among studies that assessed change in hepatic steatosis by ultrasound grading and categorization (0/1/2/3; none/mild/moderate/severe), the number of patients with mild/moderate/severe steatosis (1/2/3), at baseline who experienced improvement by ≥ 1 grade at the end of the trial was analyzed.

## 3 Study: Randomized Controlled Clinical Trials

### 3.1 Exclusion Criteria

1. The type of study was not specified.

2. Experiments conducted on animals or cells.

3. No valid outcome data could be extracted from the article.

### 3.2 Quality Assessment

Each included study was evaluated for random sequence generation, allocation hiding, blinded participants and studies, blinded outcome evaluation, incomplete outcome indicators, selective reporting, and other possible biases and classified as low risk, unclear risk, or high risk according to the Cochrane tools (Higgins 2011). Quality assessment was performed independently by Q.H. and Z.M.A., and inconsistencies were resolved through discussion by Q.F.

### 3.3 Data Extraction and Statistical Analysis

For all included outcomes, the combined effect size was expressed as odds ratio (OR), standardized mean difference (SMD), mean difference (MD), and 95% confidence interval (CI). When the measurement methods and units were identical, WMD was preferred to combine statistics. Instead, chose SMD. The classification variables were selected as risk differences and 95% CI for analysis. Data represented in medians and quartiles were converted to mean and standard deviation, which was recommended by Hozo et al. For studies that did not show changes before and after the treatment, Mean_Change_ and SD_Change_ were calculated by the method provided in the Cochrane Handbook Chapter 6.5.2 (Higgins, Li, and Deeks 2021). A correlation coefficient (Corr) of 0.5 was used in our calculations. The formula used to calculate the Mean_Change_ and SD_Change_ of the observed indicators before and after the intervention is as follows:

(1) $${\text{Mean}}_{\text{Change}}={\text{Mean}}_{\text{Final}}—{\text{Mean}}_{\text{Baseline}}.$$

(2) $$\begin{array}{c} \;\text{SDChange}\\ =\sqrt{{\text{SDFinal}}^{2}+{\text{SDBaseline}}^{2}-2\times \text{Corr}\times \text{SDFinal}\times \text{SDBaseline}.} \end{array}$$

Cochrane *Q* and *I* ^2^ tests were used to evaluate the heterogeneity. The fixed‐effects model was chosen if *I* ^2^ < 50% or *p* > 0.1; if not, the random‐effects model was selected. Subgroup analysis was conducted to explore the sources of heterogeneity, with *I* ^2^ > 50%. A sensitivity analysis was used to verify the stability of the results. Subgroup analyses were performed based on different polyphenols (curcumin, resveratrol, and silymarin) and different intervention durations (< 12 and ≥ 12 weeks). A sensitivity analysis was performed using the “one‐study‐removed” strategy to investigate the influence of each study on the effect size. The software for the meta‐analysis is Revman 5.3. A two‐tailed *p* < 0.05 was considered statistically significant.

## 4 Results

### 4.1 Characteristics of Included Studies and Quality Assessment

**FIGURE 1:**
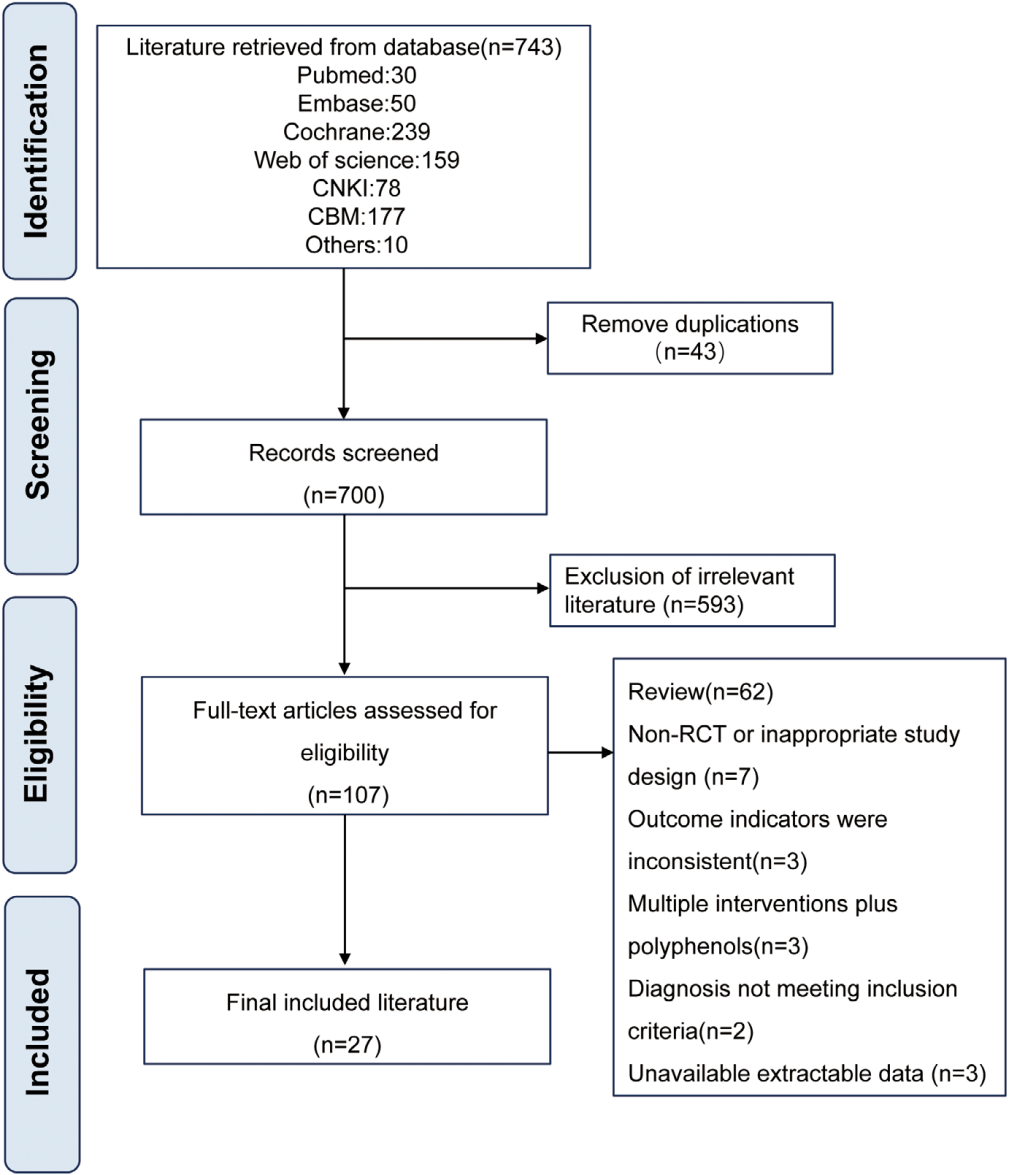
Flow diagram of the literature research.

The RCTs included in this study involved three polyphenols: curcumin, resveratrol, and silymarin. Twenty seven eligible studies met the inclusion and exclusion criteria and were included in the study (Anushiravani et al. 2019; Beheshti Namdar et al. 2023; Chachay et al. 2014; Chen et al. 2015; Cicero et al. 2020; Faghihzadeh et al. 2014; Farzin et al. 2020; Hashemi, Eskandar, and Sardabi 2009; Ghaffari et al. 2019; He et al. 2024; Heeboll et al. 2016; Jarhahzadeh et al. 2021; Jazayeri‐Tehrani et al. 2019; Kalhori et al. 2022; Kantartzis et al. 2018; Loguercio et al. 2012; Masoodi, Panahian, and Vojdanian 2013; Mirhafez, Azimi‐Nezhad, et al. 2021; Mirhafez, Dehabeh, et al. 2021; Moradi Kelardeh et al. 2020; Navarro et al. 2019; Navekar et al. 2017; Panahi et al. 2017; Panahi et al. 2019; Rahmani et al. 2016; Saadati et al. 2019; Saberi‐Karimian et al. 2020; Safari et al. 2023; Solhi et al. 2014; Wah Kheong, Nik Mustapha, and Mahadeva 2017). Some records are merged because they belong to the same RCTs. The study characteristics are listed in Table 1. The flow diagram of these studies' selection process is shown in Figure 1. There were 27 RCTs, detailed bias results are shown in Figure S1. The funnel plot generated for the reported outcomes is symmetrical. Publication bias was assessed using Egger's regression test and funnel plots when there were ≥ 10 data points (Sterne, Gavaghan, and Egger 2000).

**TABLE 1:** Characteristics of the included studies.

Polyphenols	Study	Country	Simple size (experimental/placebo)	Mean age		NAFLD diagnosis method	Duration (weeks)	Daily dosage (mg/day)	Outcome
				Experimental	Placebo				
Curcumin	Cicero et al. (2020)	Iran	40/40	54 ± 3	53 ± 5	Ultrasound	8	200	1.TC, TG, HDL‐C, LDL‐C 2.FBG, HOMA‐IR 3.BMI 4.SBP, DBP
	Kalhori et al. (2022)	Iran	21/21	40.38 ± 9.26	42.09 ± 7.23	Ultrasound	12	3000	1.Grade of hepatic steatosis 2.ALT, AST 3.TC, TG, HDL‐C, LDL‐C 4.FBG, HOMA‐IR 5.BMI 6.SBP, DBP
	Mirhafez, Azimi‐Nezhad, et al. (2021)	Iran	35/37	45.0 ± 11.1	43.1 ± 11.6	Ultrasound	8	250	1.Grade of hepatic steatosis 2.ALT, AST 3.TC, TG, HDL‐C, LDL‐C 4.FBG 5.BMI
	Moradi Kelardeh et al. (2020)	Iran	22/23	66.72 ± 3.03	64.36 ± 2.97	Ultrasound	12	80	BMI
	Panahi et al. (2017)	Iran	44/43	44.98 ± 12.59	47.21 ± 10.29	Ultrasound	8	1000	1.Grade of hepatic steatosis 2.ALT, AST 3.TC, TG, HDL‐C, LDL‐C 4, HOMA‐IR 5.BMI 6.SBP, DBP
	Panahi et al. (2019)	Iran	35/35	46.63 ± 2.21	47.51 ± 2.45	Ultrasound	12	500	1.ALT, AST 2.TC, TG, HDL‐C, LDL‐C
	Rahmani et al. (2016)	Iran	37/40	46.37 ± 11.57	48.95 ± 9.78	Ultrasound	8	500	1.Grade of hepatic steatosis 2.ALT, AST 3.TC, TG, HDL‐C, LDL‐C 4.FBG 5.BMI
	Saberi‐Karimian et al. (2020)	Iran	27/28	18–70		Ultrasound	8	500	1.Grade of hepatic steatosis 2.ALT, AST 3.TC, HDL‐C, LDL‐C 4.FBG 5.BMI 6.SBP, DBP
	Saadati et al. (2019)	Iran	27/23	46.19 ± 11.5	45.13 ± 10.9	Ultrasound/fibroscan	12	1500	1.ALT, AST 2.TC, TG, HDL‐C, LDL‐C 3.FBG, HOMA‐IR 4.BMI 5.TNF‐α
	Jarhahzadeh et al. (2021)	Iran	32/32	44.12 ± 8.35	38.56 ± 10.43	Ultrasound	12	2000	1.Grade of hepatic steatosis 2.ALT, AST 3.TC, TG, HDL‐C, LDL‐C 4.FBG
	Jazayeri‐Tehrani et al. (2019)	Iran	42/42	41.8 ± 5.6	42.5 ± 6.2	Ultrasound	12	80	1.ALT, AST 2.TC, TG, HDL‐C, LDL‐C 3.FBG, HOMA‐IR 4.BMI 5.SBP, DBP 6.TNF‐α
	Beheshti Namdar et al. (2023)	Iran	27/29	15–60		Ultrasound	8	160	ALT, AST
	Safari et al. (2023)	Iran	28/28	43.92 ± 8.74	50.35 ± 9.44	Ultrasound	12	250	1.ALT, AST 2. TC, TG, HDL‐C, LDL‐C 3. FBG 4.BMI
	He et al. (2024)	China	40/40	42 ± 10.0	40 ± 9.8	Fibro‐Touch	24	500	1.ALT, AST 2.TC, TG, HDL‐C, LDL‐C 3.FBG, HOMA‐IR 4.BMI 5.SBP, DBP
Resveratrol	Chen et al. (2015)	China	30/30	45.2 ± 10.0	43.5 ± 11.0	Ultrasound	8	300	1.Grade of hepatic steatosis 2.ALT, AST 3.TC, TG, HDL‐C, LDL‐C 4.FBG, HOMA‐IR 5.BMI 6.SBP, DBP 7.TNF‐α
	Chachay et al. (2014)	Australia	10/10	48.8 ± 12.2	47.5 ± 11.2	MRI	8	3000	1.ALT, AST 2.TC, TG, HDL‐C, LDL‐C 3.FBG, HOMA‐IR 4.BMI 5.SBP, DBP 6.TNF‐α
	Farzin et al. (2020)	Iran	25/25	39.78 ± 8.09	38.71 ± 5.76	Ultrasound	12	600	1.Grade of hepatic steatosis 2.ALT, AST 3.FBG, HOMA‐IR 4.BMI
	Faghihzadeh et al. (2014)	Iran	25/25	44.04 ± 10.10	46.28 ± 9.52	Ultrasound	12	500	1.Grade of hepatic steatosis 2.ALT, AST 3.TC, TG, HDL‐C, LDL‐C 4.FBG, HOMA‐IR 5.BMI 6.SBP, DBP 7.TNF‐α
	Heeboll et al. (2016)	Denmark	13/13	18–70		Biopsy	24	1500	1.ALT, AST 2.TG, HDL‐C, LDL‐C 3. HOMA‐IR 4.BMI 5.SBP, DBP 6.TNF‐α
	Kantartzis et al. (2018)	Denmark	53/52	18–70		MRI	12	150	1.ALT, AST 2.TC, TG, HDL‐C, LDL‐C 3. HOMA‐IR 4.SBP, DBP
Silymarin	Anushiravani et al. (2019)	Iran	30/30	47.0 ± 9.1		Ultrasound	12	140	1.ALT, AST 2.TC, TG, HDL‐C, LDL‐C 3.FBG 4.BMI
	Hashemi, Eskandar, and Sardabi (2009)	Iran	50/50	39.28 ± 11.12	39.0 ± 10.70	Ultrasound	24	280	1.ALT, AST 2.TC, TG, HDL‐C, LDL‐C 3.FBG 4.BMI
	Loguercio et al. (2012)	Italy	100/100	40.8 ± 10.3	44.2 ± 9.5	Biopsy	48	94	Grade of hepatic steatosis
	Masoodi, Panahian, and Vojdanian (2013)	Iran	50/50	48.42 ± 6.75	48.32 ± 5.45	Ultrasound	12	280	1.ALT, AST 2.BMI
	Navarro et al. (2019)	Italy	27/25	48.2 ± 11.4	49.5 ± 10.9	Biopsy	48	700	Grade of hepatic steatosis
	Solhi et al. (2014)	Iran	33/31	43.6 ± 8.3	39.36 ± 10.5	Ultrasound	8	210	ALT, AST
	Wah Kheong, Nik Mustapha, and Mahadeva (2017)	Malaysia	49/50	49.6 ± 12.7	50.1 ± 10.2	Biopsy	48	2100	1.ALT, AST 2.TC, TG, HDL‐C, LDL‐C 3.FBG, HOMA‐IR

Abbreviations: ALT, alanine aminotransferase; AST, aspartate aminotransferase; BMI, body mass index; DBP, diastolic blood pressure; FPG, fasting plasma glucose; HDL‐C, high‐density lipoprotein cholesterol; HOMA‐IR, homeo; LDL‐C, low‐density lipoprotein cholesterol; NR, not reported; SBP, systolic blood pressure; TC, total cholesterol; TG, total glyceride; TNF‐α, tumor necrosis factor‐α.

### 4.2 Effect of Polyphenols on Primary Outcomes

#### 4.2.1 Hepatic Steatosis

**FIGURE 2:**
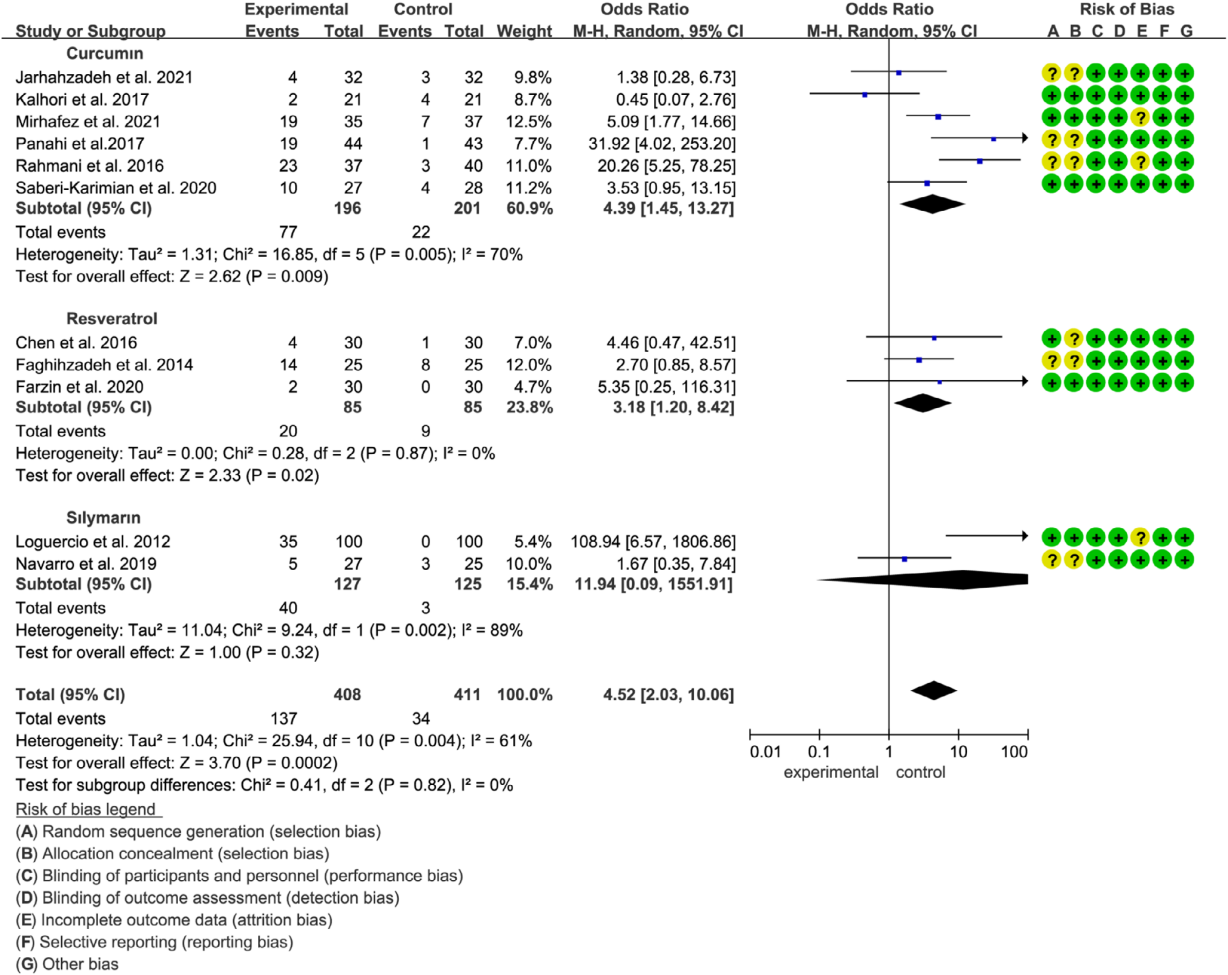
Forest plots of the effect of polyphenols on improvement in hepatic steatosis.

The effect of curcumin, resveratrol, and silymarin on improvement in hepatic steatosis, as graded by liver ultrasound or histology, was examined across 11 studies involving a total of 819 participants (408 in the polyphenol group and 411 in the placebo group). The combined effect of three specific polyphenols—curcumin, resveratrol, and silymarin—on hepatic steatosis improvement was statistically significant (OR: 4.52, 95% CI: 2.03 to 10.06, *p* = 0.0002, *I* ^2^ = 61%). Subgroup analysis revealed that curcumin exhibited the most pronounced effect in enhancing the odds of improvement from mild, moderate, or severe hepatic steatosis compared to placebo (OR: 4.39, 95% CI: 1.45 to 13.27, *p =* 0.009) followed by resveratrol (OR: 3.18, 95% CI: 1.20 to 8.42, *p =* 0.02) (Figure 2). Further analysis indicated that polyphenols were particularly effective in improving hepatic steatosis when the intervention period was less than 12 weeks (OR: 7.94, 95% CI: 3.48 to 18.10, *p* < 0.00001) (Figure S2A). Sensitivity analysis confirmed the robustness of the overall effect size.

#### 4.2.2 Alanine Aminotransferase Activity (ALT)

**FIGURE 3:**
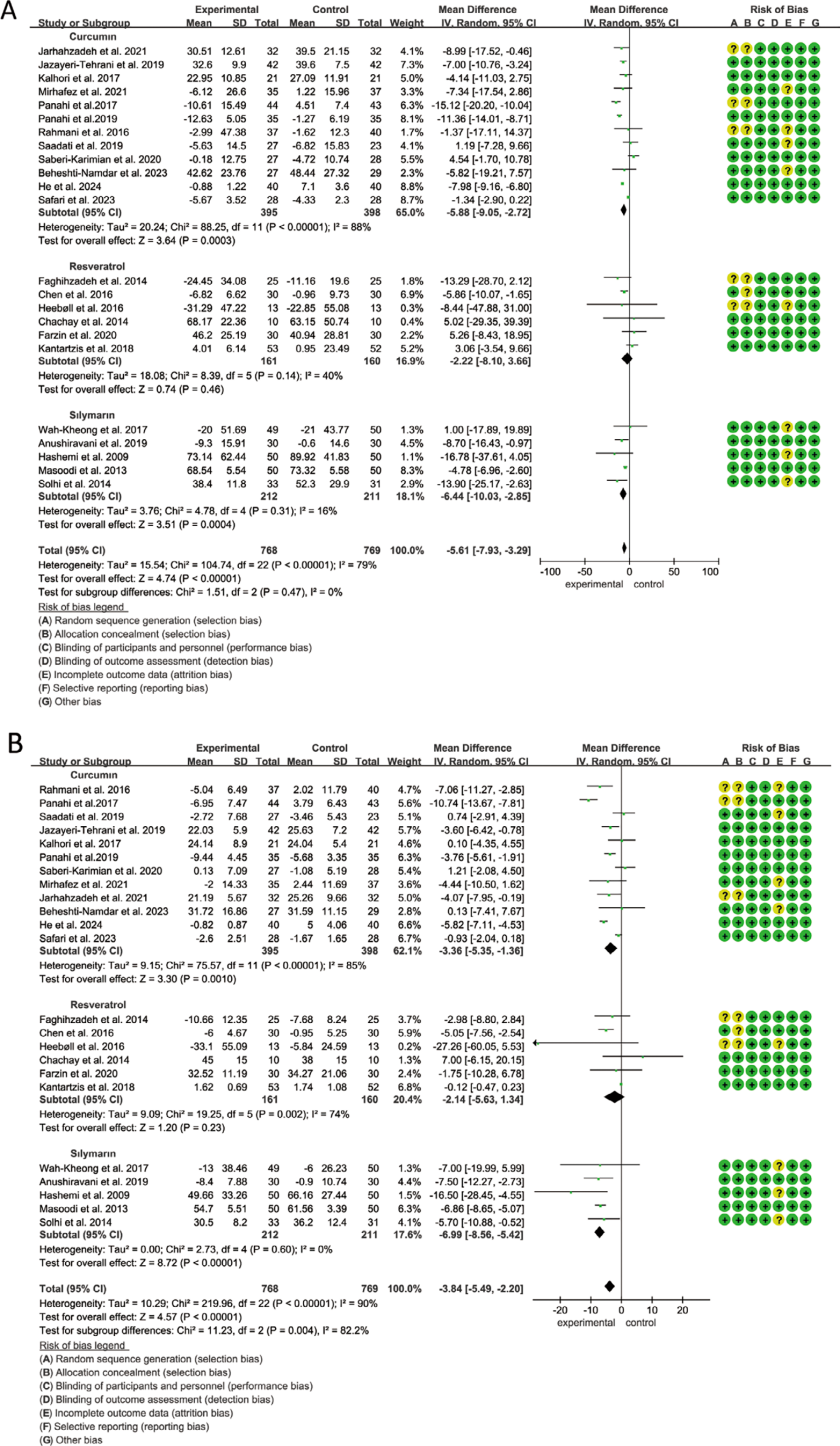
Forest plots of the effect of polyphenols on transaminase activity, including (A) ALT and (B) AST. ALT, alanine transaminase; AST, aspartate aminotransferase.

A total of 23 studies, involving 1537 participants (768 in the polyphenol group and 769 in the placebo group), were included to evaluate the impact of curcumin, resveratrol, and silymarin on ALT, and heterogeneity tests (*p* < 0.01, *I* ^2^ = 79%) suggested statistical heterogeneity among studies. The findings demonstrated that curcumin, resveratrol, and silymarin notably reduced ALT levels (MD: −5.61 U/L, 95% CI: −7.93, −3.29, *p* < 0.00001), as analyzed using a random‐effects model (Figure 3A). Subgroup analysis indicated that silymarin had the most substantial effect in lowering ALT levels (MD: −6.44 U/L, 95% CI: −10.03 to −2.85, *p* = 0.0004) followed by curcumin (MD: −5.88 U/L, 95% CI: −9.05 to −2.72, *p* = 0.0003) (Figure 3A). Additionally, subgroup analyses revealed that polyphenols significantly reduced liver enzyme levels whether the treatment duration exceeded 12 weeks or not (Figure S2B). Sensitivity analysis confirmed that the overall effect size remained largely consistent.

### 4.3 Effect of Polyphenols on Secondary Outcomes

#### 4.3.1 Aspartate Aminotransferase Activity (AST)

The same 23 studies as ALT, evaluated the impact of curcumin, resveratrol, and silymarin on AST levels, revealing considerable inter‐study heterogeneity (*p* < 0.01, *I* ^2^ = 90%). Our analysis, using a random‐effects model, demonstrated that polyphenols significantly lowered AST levels (MD: −3.84 U/L, 95% CI: −5.49 to −2.20, *p* < 0.00001) (Figure 3B). Subgroup analyses further indicated a marked reduction in AST with silymarin (MD: −6.99 U/L, 95% CI: −8.56 to −5.42, *p* < 0.00001), followed by curcumin (MD: −3.36 U/L, 95% CI: −5.35 to −1.36, *p* < 0.00001). In contrast, resveratrol did not show a statistically significant effect (MD: −2.14 U/L, 95% CI: −5.63 to 1.34, *p* = 0.23) (Figure 3B). Furthermore, the results suggested significant differences in the magnitude of AST reduction across varying intervention durations (Figure S2C). Sensitivity analysis confirmed the stability of the overall effect size.

#### 4.3.2 Blood Lipid Indices (TC, TG, HDL‐C, and LDL‐C)

**FIGURE 4:**
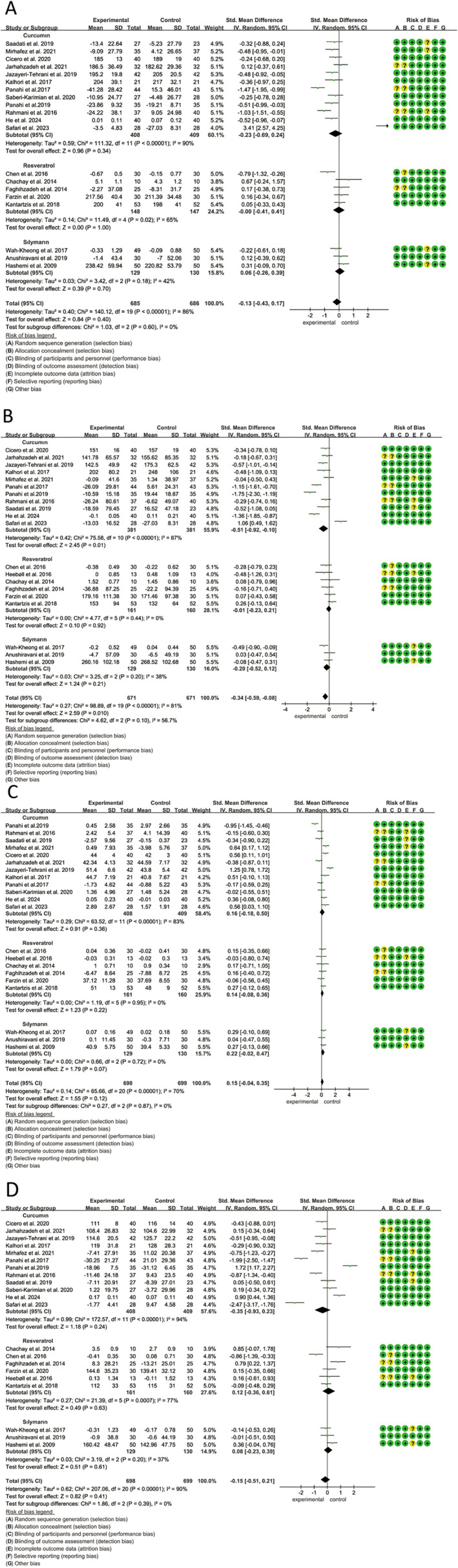
Forest plots of the effect of polyphenols on blood lipid indices, including (A) TC, (B) TG, (C) HDL‐C, and (D) LDL‐C. HDL‐C, high‐density lipoprotein cholesterol; LDL‐C, low‐density lipoprotein cholesterol; TC, total cholesterol; TG, total glyceride.

A total of 20 studies, encompassing 1371 participants (685 receiving polyphenols and 686 on placebo), examined the effects of curcumin, resveratrol, and silymarin on TC in individuals with MASLD. The analysis indicated that polyphenol intake had no significant impact on TC levels (SMD: −0.13, 95% CI: −0.43 to −0.17) (*p* = 0.40, *I* ^2^ = 86%) (Figure 4A). Another subgroup analysis showed that polyphenols significantly reduced TC when the intervention period was less than 12 weeks (SMD: −0.57, 95% CI: −1.00 to −0.14, *p* = 0.009) (Figure S2D). Moreover, sensitivity analysis demonstrated that excluding any individual study had minimal impact on the overall heterogeneity.

Twenty studies, including 1342 participants (671 polyphenols users and 671 placebo users), reported the effects of three polyphenols (curcumin, resveratrol, and silymarin) on TG. Subgroup analysis indicated that curcumin supplementation significantly reduced TG levels (SMD: −0.51, 95% CI: −0.92 to −0.10, *p* = 0.01, *I* ^2^ = 87%) based on the random‐effects model (Figure 4B). Furthermore, the significant differences were observed in TG level changes while intervention durations less than 12weeks (Figure S2E). Sensitivity analysis showed that excluding any reference had little effect on overall heterogeneity (Figure S4).

A total of 21 studies, involving 1397 participants (698 in the polyphenol group and 699 in the placebo group), provided data on HDL‐C levels. The analysis showed that curcumin, resveratrol, and silymarin did not result in significant improvements in HDL‐C (SMD: 0.15, 95% CI: −0.04 to 0.35, *p* = 0.12) according to the random‐effects model (*p* < 0.01, *I* ^2^ = 70%) (Figure 4C). The effect of different polyphenols and intervention durations on HDL‐C levels in MASLD patients was similarly insignificant (Figure S2F). Sensitivity analysis revealed that the effect of silymarin on HDL‐C levels in MASLD patients became significant (SMD: 0.28, 95% CI: 0.00 to 0.56, *p* = 0.05) after excluding the study by (Anushiravani et al. 2019).

Similarly, in the same population as the HDL‐C analysis, it was found that curcumin, resveratrol, and silymarin had no significant effect on LDL‐C levels (SMD: −0.15, 95% CI: −0.51 to 0.21, *p* = 0.41) using the random‐effects model (*p* < 0.01, *I* ^2^ = 90%) (Figure 4D). However, subgroup analysis indicated that polyphenols significantly reduced LDL‐C levels when the intervention period was less than 12 weeks (Figure S2G). Sensitivity analysis further demonstrated that, upon removing the study by Panahi et al. curcumin significantly lowered LDL‐C levels in MASLD patients (SMD: −0.50, 95% CI: −0.92 to −0.07, *p* = 0.02).

#### 4.3.3 Blood Glucose Indicators (FBG and HOMA‐IR)

**FIGURE 5:**
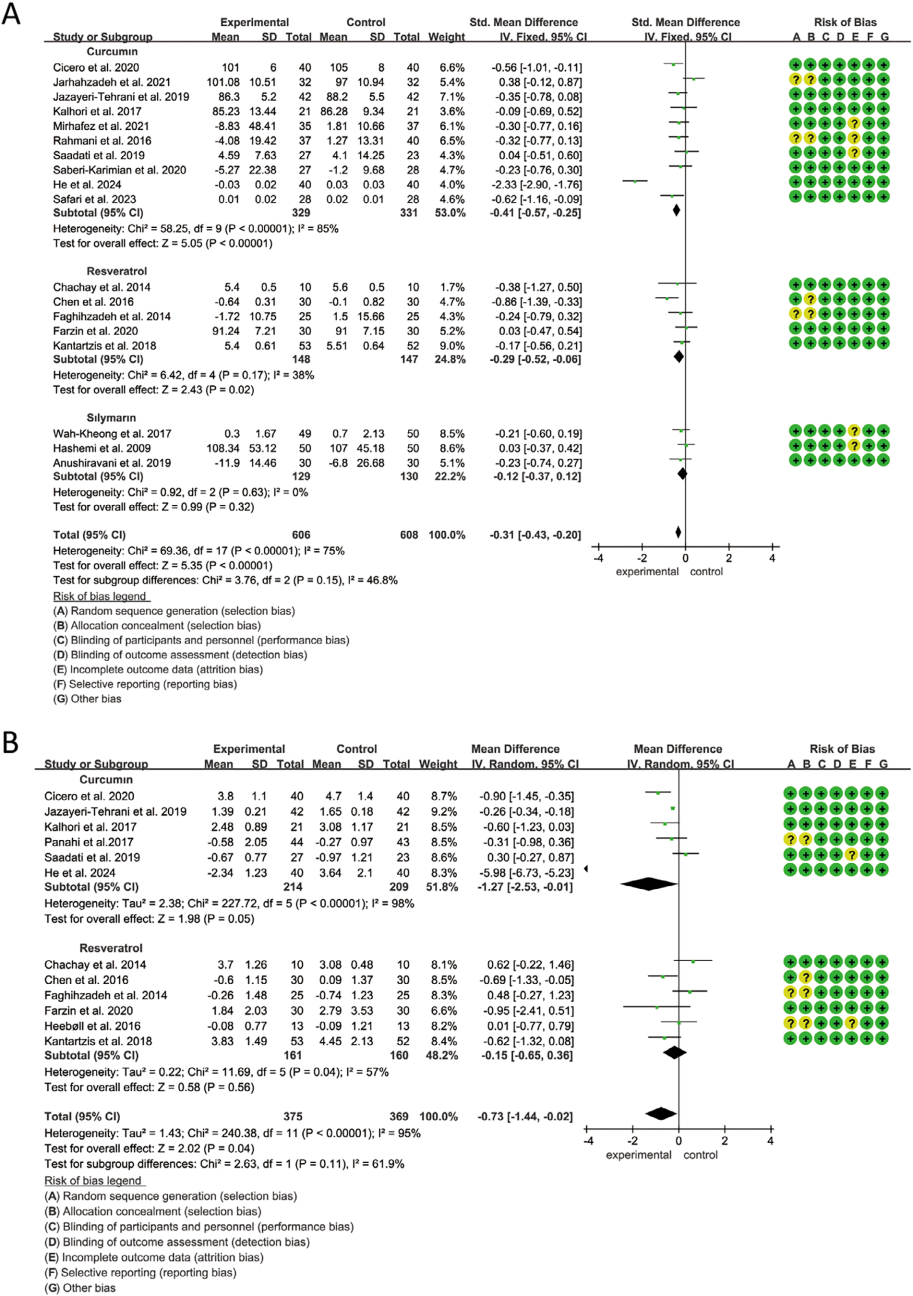
Forest plots of the effect of polyphenols on blood glucose indicators, including (A) FBG and (B) HOMA‐IR. FPG, fasting plasma glucose; HOMA‐IR, homeostasis model assessment‐insulin resistance.

Based on the analysis of 18 effect sizes from studies involving 1214 participants (606 in the polyphenol group and 608 in the placebo group), changes in fasting blood glucose (FBG) were assessed using a fixed‐effects model, in accordance with the results of the heterogeneity test (*p* < 0.01, *I* ^2^ = 75%). The findings indicated that curcumin, resveratrol, and silymarin significantly reduced FBG levels in patients with MASLD (SMD: −0.31, 95% CI: −0.43 to −0.20, *p* < 0.0001) (Figure 5A). Subgroup analyses revealed that curcumin had the most pronounced effect on lowering FBG (WMD: −0.41, 95% CI: −0.57 to −0.25, *p* < 0.001), followed by resveratrol (WMD: −0.29, 95% CI: −0.52 to −0.06, *p* = 0.02), while silymarin did not demonstrate a significant effect (WMD: −0.12, 95% CI: −0.37 to 0.12, *p* = 0.32) (Figure 5A). Moreover, polyphenols were particularly effective in reducing FBG levels when the intervention period exceeded 12 weeks (Figure S2H). Sensitivity analyses confirmed the robustness of the results, as no significant changes were observed upon the exclusion of any individual study.

Twelve studies, encompassing 744 participants (375 polyphenol users and 369 placebo users), reported on changes in HOMA‐IR before and after treatment. Analysis of 12 effect sizes indicated a significant impact of curcumin, resveratrol, and silymarin on HOMA‐IR in patients with MASLD (MD: −0.73, 95% CI: −1.44 to −0.02, *p* = 0.04, *I* ^2^ = 95%) (Figure 5B). Subgroup analysis revealed that curcumin had a significant effect on reducing HOMA‐IR (MD: −1.27, 95% CI: −2.53 to −0.01, *p* = 0.05), while resveratrol did not show significant differences between the treatment and control groups (MD: −0.15, 95% CI: −0.65 to 0.36, *p* = 0.56) (Figure 5B). The intervention duration had little effect on blood pressure (Figure S2I). Silymarin was involved in only one study, so it was not subjected to subgroup analysis. Sensitivity analysis confirmed that the overall effect size remained consistent, with no significant changes observed upon the exclusion of any individual study.

#### 4.3.4 Body Mass Index (BMI)

**FIGURE 6:**
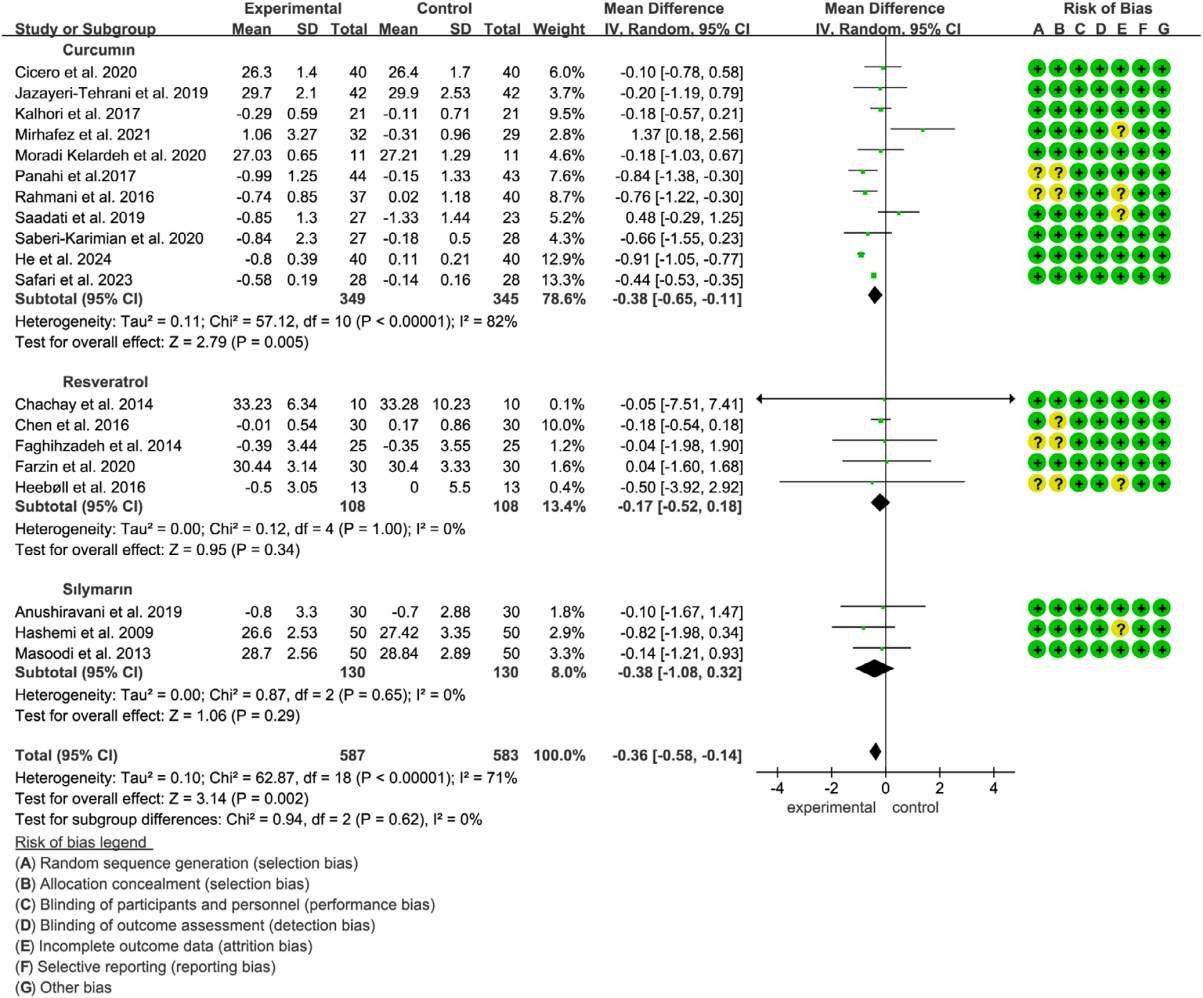
Forest plots of the effect of polyphenols on BMI. BMI, body mass index.

Based on a random‐effects model (*p* < 0.001, *I* ^2^ = 71%), an analysis of 19 studies involving 1170 participants (587 in the polyphenol group and 583 in the placebo group) demonstrated that curcumin, resveratrol, and silymarin significantly reduced BMI (MD: −0.36, 95% CI: −0.58 to −0.14, *p* = 0.002) (Figure 6). Subgroup analysis revealed that curcumin contributed to a significant reduction in BMI (MD: −0.35, 95% CI: −0.65 to −0.04, *p* = 0.02) (Figure 6), particularly in studies with an intervention period exceeding 12 weeks (Figure S2J). Sensitivity analyses confirmed the robustness of these findings, as no significant changes occurred upon the exclusion of any single study.

#### 4.3.5 Blood Pressure (SBP and DBP)

**FIGURE 7:**
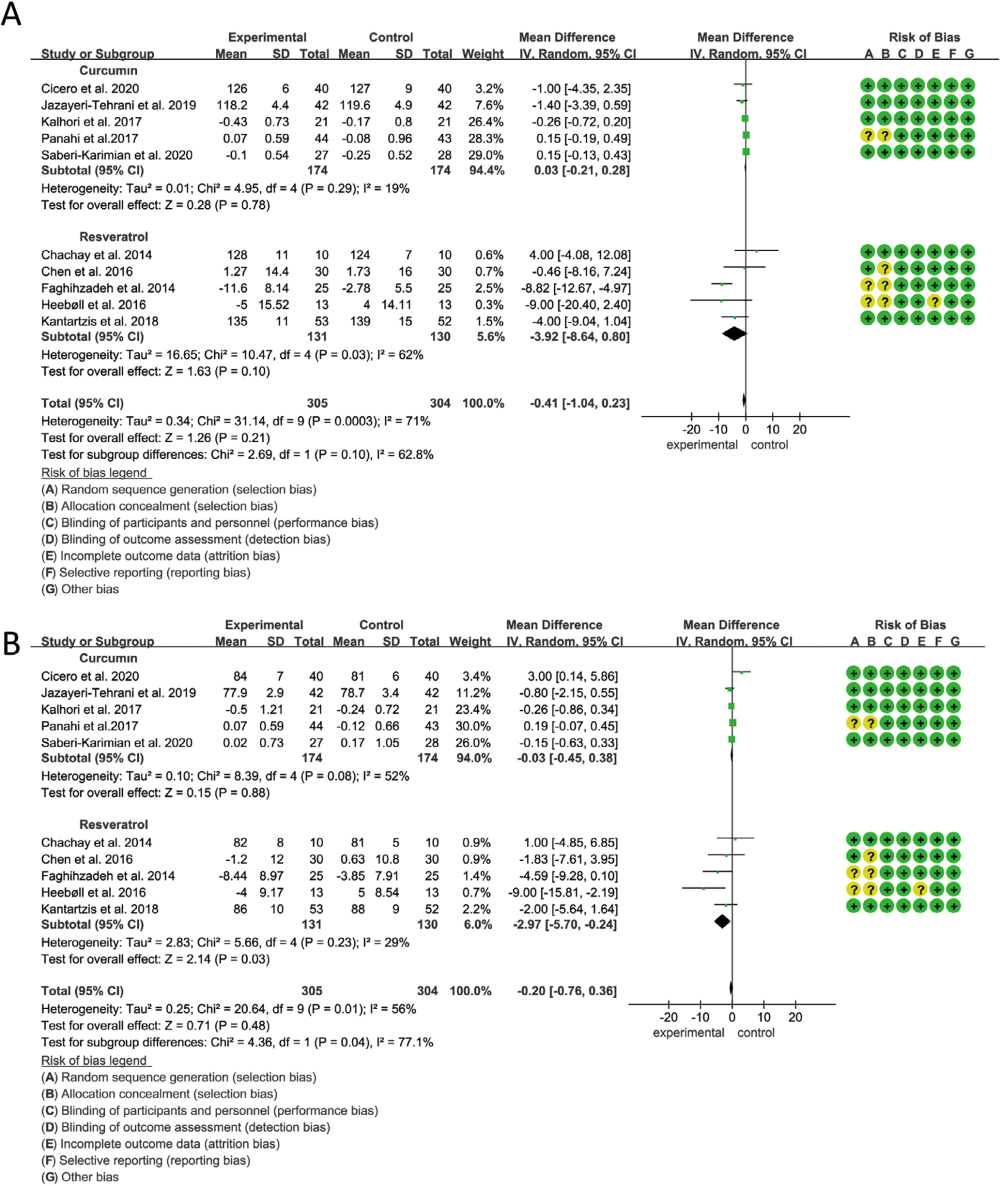
Forest plots of the effect of polyphenols on blood pressure, including (A) SBP and (B) DBP. SBP: systolic blood pressure. DBP, diastolic blood pressure.

Ten studies, comprising 609 participants (305 in the polyphenol group and 304 in the placebo group), evaluated the effects of curcumin, resveratrol, and silymarin on blood pressure. The analysis indicated that polyphenol intake did not significantly affect systolic blood pressure (SBP), with a weighted mean difference (WMD) of −0.41 mmHg (95% CI: −1.04 to 0.23, *p* = 0.21), according to the random‐effects model (*p* < 0.01, *I* ^2^ = 71%) (Figure 7A). However, sensitivity analysis revealed that upon excluding the study by (Faghihzadeh et al. 2014), heterogeneity decreased to 38%, and resveratrol was found to significantly reduce SBP (MD: −5.78 mmHg, 95% CI: −9.67 to −1.90, *p* = 0.004). Additionally, resveratrol intake was associated with a significant reduction in DBP (MD: −2.97 mmHg, 95% CI: −5.70 to −0.24, *p* = 0.03) (Figure 7B). A significant improvement in SBP was observed in interventions lasting longer than 12 weeks (Figure S2K,L). Sensitivity analyses confirmed the robustness of the results, with no significant changes detected after excluding any individual study. Notably, no studies assessed the impact of silymarin on blood pressure in this context.

#### 4.3.6 Inflammatory Factor (TNF‐α)

**FIGURE 8:**
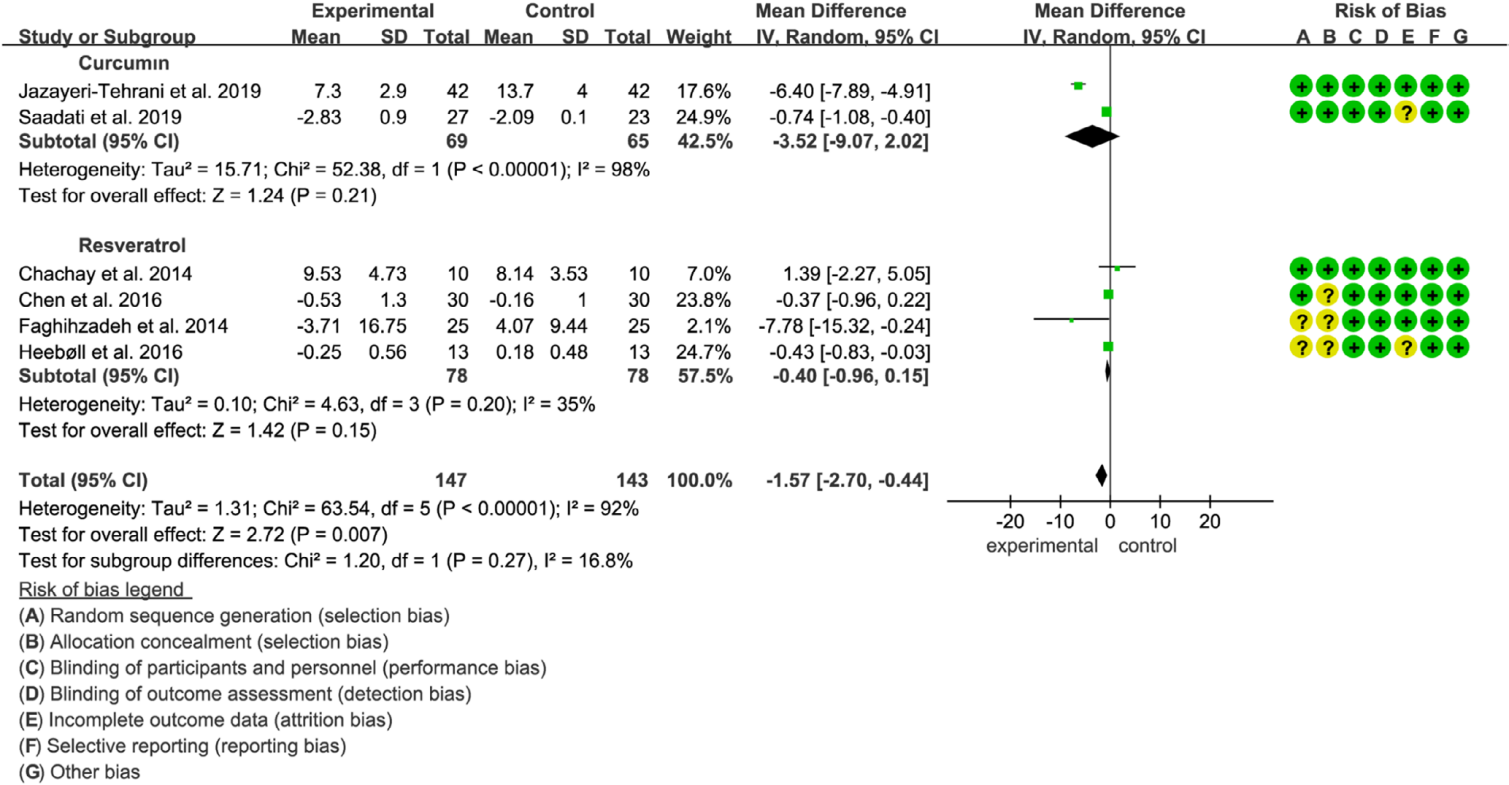
Forest plots of the effect of polyphenols on inflammatory factor (TNF‐α). TNF‐α, tumor necrosis factor‐α.

Six studies included290 participants (147 polyphenols users and 143 placebo users). We found that curcumin, resveratrol, and silymarin significantly reduced TNF‐α (MD: −1.57 pg/mL, 95% CI: −2.70, −0.44, *p* = 0.007) (Figure 8) based on the random‐effects model while the intervention duration more than 12 weeks (Figure S2M). However, there was no significant difference in individual subgroup (Figure 8). The overall effect size remained unchanged after performing sensitivity analysis. No study assessed the impact of resveratrol in this regard.

### 4.4 Publication Bias

To identify the publication bias among the eligible studies, the Egger test was utilized. Publication bias analysis did not highlight any differences between the observed and estimated values (*p* > 0.05) (Figure S3).

## 5 Discussion

### 5.1 Principal Findings

In this meta‐analysis, we systematically evaluated the effects of the three polyphenols (curcumin, resveratrol, and silymarin) on hepatic steatosis, transaminase levels, blood lipids, and other related indicators in patients with MASLD. The results demonstrated that these polyphenols were associated with improvements in hepatic steatosis, glycemic control, lipid metabolism, and inflammation. Subgroup analysis revealed that curcumin had the most pronounced effect in reducing hepatic steatosis, along with significant improvements in transaminase levels and various metabolic markers. Silymarin showed the strongest effect in lowering transaminase levels. While not the strongest in any specific outcomes, resveratrol still was associated with significant improvements in hepatic steatosis and metabolic parameters.

### 5.2 Potential Mechanisms

**FIGURE 9:**
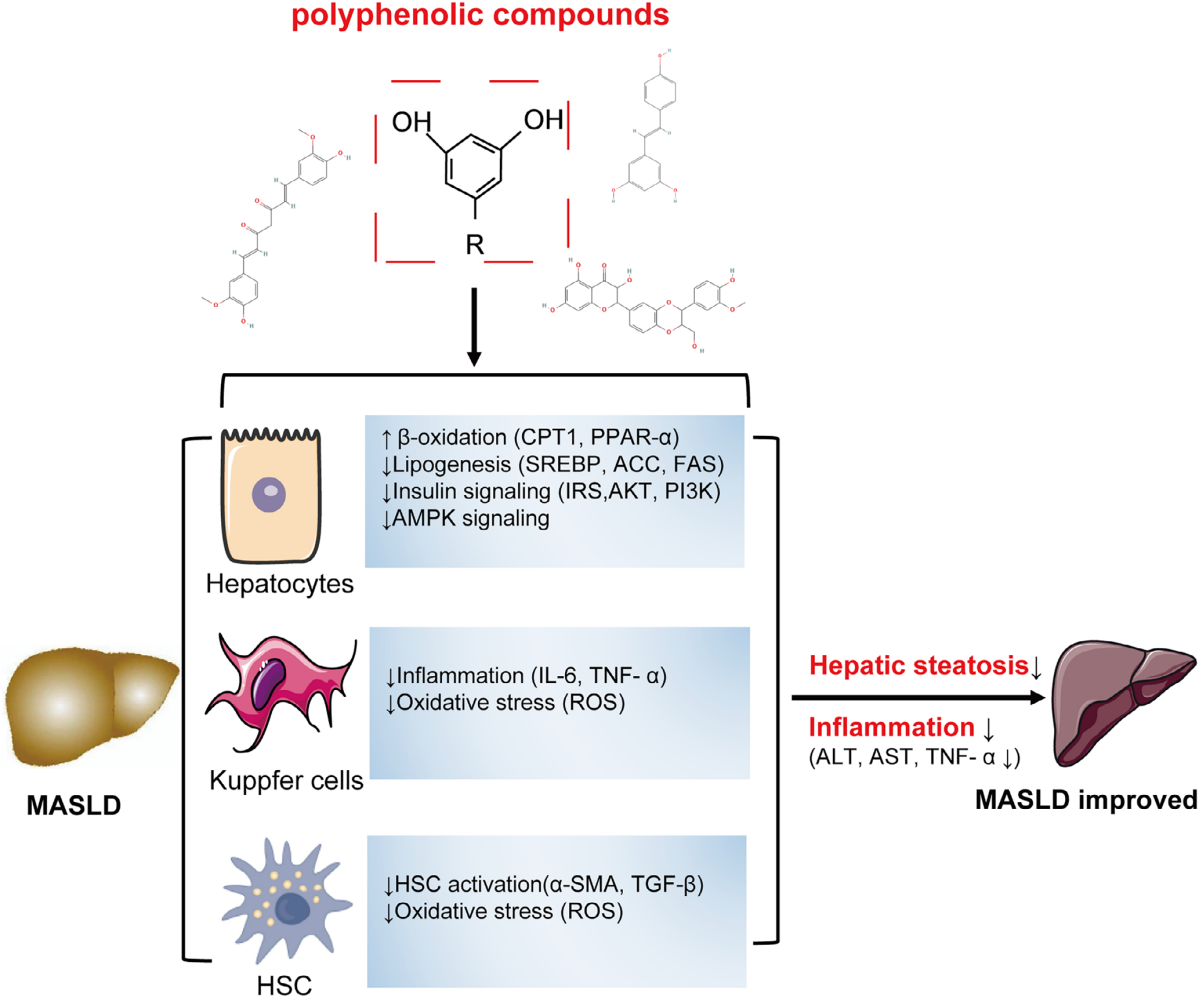
Molecular effects of polyphenols on MASLD. (1) In the hepatocytes, polyphenols inhibit lipogenesis and promote FA oxidation and stimulate insulin (IRS/PI3K/AKT) and AMPK signaling; (2) Inhibition of the activation of HSC, reduce the production of ROS and fibrogenic cytokines; (3) Inhibition of ROS and inflammatory cytokines production in Kupfer cells. MASLD: Non‐alcoholic fatty liver disease, FA: fatty acid, IRS/PI3K/AKT: Insulin receptor substrate 1/phosphatidylinositol‐3‐kinase/protein kinase B. AMPK, AMP‐activated protein kinase; HSC, hepatic stellate cells; ROS, reactive oxygen species.

Polyphenols have a common anti‐MASLD mechanism, ranging from lipogenesis regulation to modulation of insulin resistance, oxidative stress modification, and inflammation control (Prochazkova, Bousova, and Wilhelmova 2011); (Rafiei, Omidian, and Bandy 2017), (Figure 9). In terms of specific molecular mechanisms, (Kang et al. 2013). found that curcumin activates AMP‐activated protein kinase (AMPK) and inhibits sterol regulatory element‐binding protein (SREBP‐1c) and fatty acid synthase expression in hepatocytes, leading to reduced hepatic lipogenesis. Another study found that the inhibitory effect of curcumin on HSC activation depends on blocking of NF‐kB and ERK signaling (Chen and Zheng 2008) and inducing of PPAR‐γ (Lin et al. 2012). Several studies have shown that resveratrol can also activate AMPK and regulate SIRT1 to improve hepatic lipid metabolism (Baur et al. 2006; Lagouge et al. 2006). At the same time, it can treat experimental NASH by inhibiting fat synthesis [down‐regulating SREBP‐1, fatty acid synthase (FAS), and acetyl‐CoA carboxylase (ACC)], promoting fat acid oxidation (up‐regulation of CPT‐1 and ACO) (Alberdi et al. 2013; Gomez‐Zorita et al. 2012), and regulating intestinal microflora (Heeboll et al. 2015; Lagouge et al. 2006; Shang et al. 2008); (Salamone et al. 2012) and (Zhang et al. 2013) confirmed that silymarin can inhibit the activity of nuclear factor‐κB (NF‐κB) and regulate IRS‐1/PI3K/AKT pathway to alleviate IR. Different polyphenols, such as resveratrol and curcumin, exert their effects through similar molecular targets acting on the AMPK pathway, suggesting that these compounds may share the same molecular pathway in lipid metabolism (Cheng et al. 2020). In addition, curcumin and silymarin both act on the NF‐κB pathway to relieve inflammation, indicating that they may share the same molecular mechanism in the inflammatory response. We summarized 16 RCT studies of polyphenolic compounds in the treatment of MASLD through systematic review and meta‐analysis to provide the latest clinical evidence for clinicians and patients in the future.

### 5.3 Comparison With Other Studies

Previous studies reviewed the effects of polyphenols on MASLD, with curcumin, resveratrol, and silymarin being the most examined compounds (Bayram, Majoo, and Ozturkcan 2021). In line with these findings, we conducted a meta‐analysis that utilized more objective data and employed rigorous methodologies to further validate the therapeutic effects of curcumin, resveratrol, and silymarin.

In addition, Yang et al. conducted a meta‐analysis evaluating the effects of polyphenols on MASLD but did not exclude studies involving combination therapies, nor did they compare the efficacy of different polyphenols (Yang et al. 2022). To address these limitations, our meta‐analysis adopted a more stringent approach by focusing exclusively on placebo‐controlled studies where polyphenols were used as the sole intervention. Additionally, we conducted subgroup analyses to directly compare the therapeutic efficacy of curcumin, resveratrol, and silymarin. This careful selection allowed us to provide more definitive and reliable evidence on the efficacy of polyphenols in the management of MASLD.

Hepatic steatosis is a hallmark of MASLD (Younossi et al. 2018). Previous studies suggested that polyphenolic compounds may reduce hepatic fat accumulation (Bayram, Majoo, and Ozturkcan 2021; Peng et al. 2020). However, to our best knowledge, this is the first meta‐analysis of published randomized controlled trials to comprehensively evaluate the effectiveness of polyphenolic compounds on hepatic steatosis in MASLD patients. The eleven RCTs included in our analysis showed that the overall effect of the three polyphenols—curcumin, resveratrol, and silymarin—significantly increased the odds of improvement from mild, moderate, or severe hepatic steatosis. Curcumin supplementation improved MASLD severity as assessed by liver ultrasonography findings, which is consistent with recent meta‐analyses of curcumin's effect on MASLD (Ngu et al. 2022). Different from other meta‐analyses, we further found that curcumin had the most pronounced effect on improving hepatic steatosis, followed by resveratrol based on the subgroup results.

The decision to use ALT and AST levels as primary outcomes was because these are main markers of hepatocellular injury, which is more pronounced in patients with MASLD (Giannini, Testa, and Savarino 2005). The effects of some polyphenolic compounds on liver enzymes appear to be inconsistent. Our study indicated that curcumin supplementation reduced AST and ALT levels, which is consistent with recent reports (Ebrahimzadeh et al. 2024; Molani‐Gol, Dehghani, and Rafraf 2024). In the meta‐analysis conducted by Zhang et al. resveratrol was demonstrated not to affect the activity of liver enzymes (Zhang et al. 2016). In turn, a study focusing on patients with MASLD showed that a six‐month therapy with resveratrol supplementation contributed to decreasing the levels of hepatic enzymes (Jakubczyk et al. 2020; Wei and Yu 2021). In the present study, it was demonstrated that the decline in ALT following resveratrol treatment in MASLD patients was not statistically significant. These discrepancies may be attributed to differences in resveratrol dosage, treatment duration, and patient characteristics across studies. The previous study found that silymarin has anti‐inflammatory, immunomodulatory, antifibrotic, antioxidant, and liver regeneration properties in the treatment of MASLD (Abenavoli et al. 2018; Salvoza et al. 2022). Our results showed that silymarin had the strongest effect on decreasing ALT and AST followed by curcumin.

Cardiovascular diseases (CVDs) are the leading cause of death in patients with MASLD (Targher, Byrne, and Tilg 2020). One reason for the development of CVDs is atherogenic dyslipidemia, characterized by low HDL‐C and high LDL‐C, TC, and TG levels. Our subgroup analysis found that curcumin significantly reduced TG compared with placebo, whereas resveratrol and silymarin did not show significant effects. This finding is consistent with previous studies suggesting that curcumin has lipid‐lowering effects (Sahebkar et al. 2016).

In terms of glucose metabolism, our results showed that resveratrol and curcumin reduced fasting blood glucose (FBG) levels; additionally, curcumin had a significant effect on HOMA‐IR. These findings are consistent with previous meta‐analyses (Jakubczyk et al. 2020; Molani‐Gol, Dehghani, and Rafraf 2024). The hypoglycemic effects of curcumin may be attributed to its ability to enhance insulin sensitivity and modulate inflammatory pathways. This meta‐analysis found that curcumin significantly reduced BMI. This is similar to previously published studies showing that polyphenols have anti‐obesity properties that can increase energy expenditure and lipolysis (Boccellino and D'Angelo 2020).

### 5.4 Strengths and Limitations

Our study has several notable strengths. First, a key strength lies in the direct comparison of the effects of polyphenolic compounds in patients with MASLD, providing a clear assessment of their efficacy. Second, we exclusively included randomized controlled trials (RCTs), which are considered the gold standard for evaluating intervention effectiveness. The use of randomization minimizes selection bias, enhancing the reliability of the pooled results (Ijaz et al. 2014). Third, the literature search was comprehensive, making the conclusions credible.

Our study is not without limitations. First, although no statistical publication bias was found, regional and publication biases remain a concern, as most studies were conducted in Iran, and potential unpublished studies may limit generalizability. Additionally, bias detection methods have limitations (Loomba and Sanyal 2013), underscoring the need for larger, more diverse studies to confirm these findings across different populations. Second, the sensitivity analysis revealed both variability in study quality and significant heterogeneity in some outcomes, which could affect the robustness of our conclusions. Third, only a limited number of studies provided data from sequential liver biopsies conducted before and after treatment, restricting our ability to fully assess treatment efficacy over time. Despite these shortcomings, we believe that our results support the consideration of well‐designed and larger studies to assess polyphenols as a treatment option for MASLD.

### 5.5 Clinical Implications

This study has implications for future clinical practice. Hepatic steatosis, with elevated transaminase levels, is the most common clinical manifestation of MASLD. Though most studies used ALT, AST, and B‐ultrasound not liver biopsy to evaluate the efficacy of drugs, this meta‐analysis showed that curcumin, resveratrol, and silymarin can effectively improve liver steatosis, reduce serum transaminase activity, and inhibit inflammation. The finding will push for the creation of new trials whose solid endpoints are the histological improvement. And these results suggest polyphenol may be a promising complementary and alternative therapy for MASLD.

### 5.6 Conclusion

The current meta‐analysis suggests that curcumin, resveratrol, and silymarin offer some benefits in treating MASLD. Among the polyphenols studied, curcumin shows the most promise in reducing hepatic steatosis, silymarin has the strongest effect on lowering transaminase levels. While not the strongest in any specific outcomes, resveratrol still was associated with significant improvements in steatosis and metabolic parameters. However, due to the potential biases and limitations in the included studies, further high‐quality research, particularly with larger cohorts of biopsy‐proven MASLD patients, is needed to confirm these findings.

## Author Contributions

**Qian Huang:** data curation (equal), formal analysis (equal), investigation (equal), writing – original draft (lead). **Ziming An:** investigation (equal). **Xin Xin:** investigation (equal). **Xiaojun Gou:** funding acquisition (equal). **Xiaoting Tian:** writing – review and editing (equal). **Yiyang Hu:** writing – review and editing (equal). **Zubing Mei:** methodology (equal). **Qin Feng:** funding acquisition (equal), methodology (equal), writing – review and editing (equal).

## Conflicts of Interest

The authors declare no conflicts of interest.

## Supporting information


Data S1.

## Data Availability

Data can be available upon request.
