# Supplementary material for: The Effectiveness of Curcumin, Resveratrol, and Silymarin on MASLD: A Systematic Review and Meta‐Analysis
Source: Food Sci Nutr. 2024 Nov 14;12(12):10010–29. doi: 10.1002/fsn3.4595 (PMC11666838; doi:10.1002/fsn3.4595)
Supplement: Supplementary file 1 — Data S1. [file FSN3-12-10010-s001.docx]

***Supplementary Material***

**The Effectiveness of** **Curcumin, Resveratrol, and Silymarin on MASLD: A Systematic Review and** **Meta-Analysis**

Qian Huang^1a, b^, Ziming An^1a^, Xin Xin^1a^, Xiaojun Gou^2^, Xiaoting Tian^3^, Yiyang Hu^1a^, Zubing Mei^4*^, Qin Feng^1a,5,6,7*^

1a Institute of Liver Diseases, Shuguang Hospital Affiliated to Shanghai University of Traditional Chinese Medicine, Shanghai, China; 1b School of Basic Medicine, Shaanxi University of Traditional Chinese Medicine, Shaanxi, China,

2Central Laboratory, Baoshan District Hospital of Integrated Traditional Chinese and Western Medicine of Shanghai, Shanghai, China,

3Shanghai Institute of Materia Medica,Chinese Academy of Sciences, Shanghai, China,

4Institute of Anorectal Diseases, Shuguang Hospital Affiliated to Shanghai University of Traditional Chinese Medicine, Shanghai, China,

5Shanghai Key Laboratory of Traditional Chinese Clinical Medicine, Shanghai, China,

6Key Laboratory of Liver and Kidney Diseases, Shanghai University of Traditional Chinese Medicine, Ministry of Education, Shanghai, China

*Correspondence:

Qin Feng: fengqin@shutcm.edu.cn

Zubing Mei: herrmayor@shutcm.edu.cn

**P****2: Figure S1 Quality assessment**

**P3-9: Figure S2 Forest plots of subgroups.**

**P9-10: Figure S3 Publication bias**

**P11-14: Figure S4 Search strategy**

**Figure S1 A. The methodological quality of included studies on effect of** **polyphenols intervention based on review authors’ judgments about each risk of bias item presented as percentages across all included studies.**

**Figure S1B. The methodological quality of included studies on effect of polyphenols intervention based on review authors’ judgments about each risk of bias item for each included study.**

**Figure S2. Forest plots of subgroups analysis to polyphenols supplementation by two different intervention duration (＜12w and ≥12w).**

(A)Improvement of hepatic steatosis

(B)ALT (U/L)

(C)AST (U/L)

(D)TC

(E)TG

(F)HDL-C

(G)LDL-C

(H)FBG

(I)HOMA-IR

(J)BMI (kg/m^2^)

(K)SBP（mmHg）

(L)DBP（mmHg）

(M)TNF

**Figure S3. Publication bias assessed with funnel plots and Egger's test.**


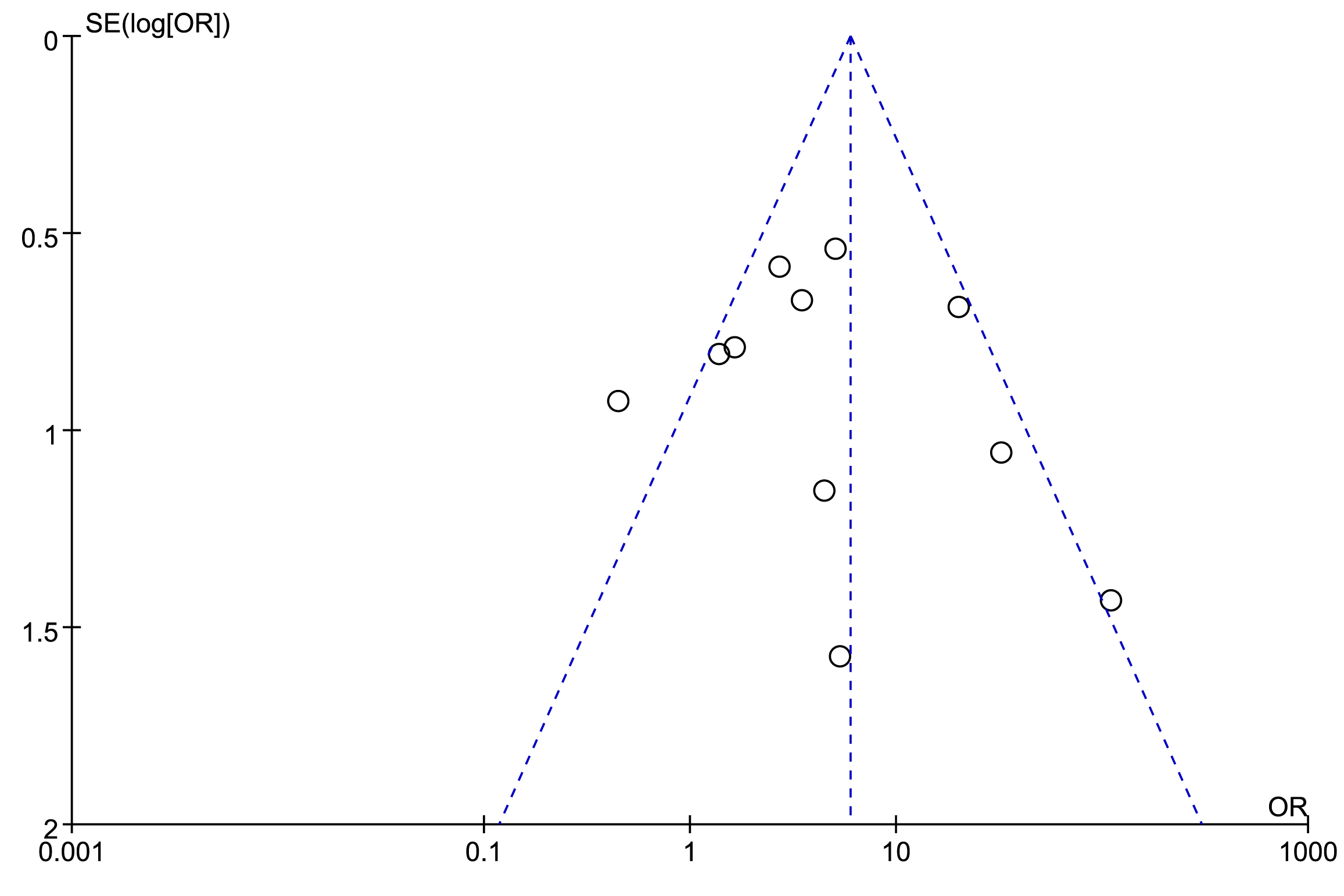

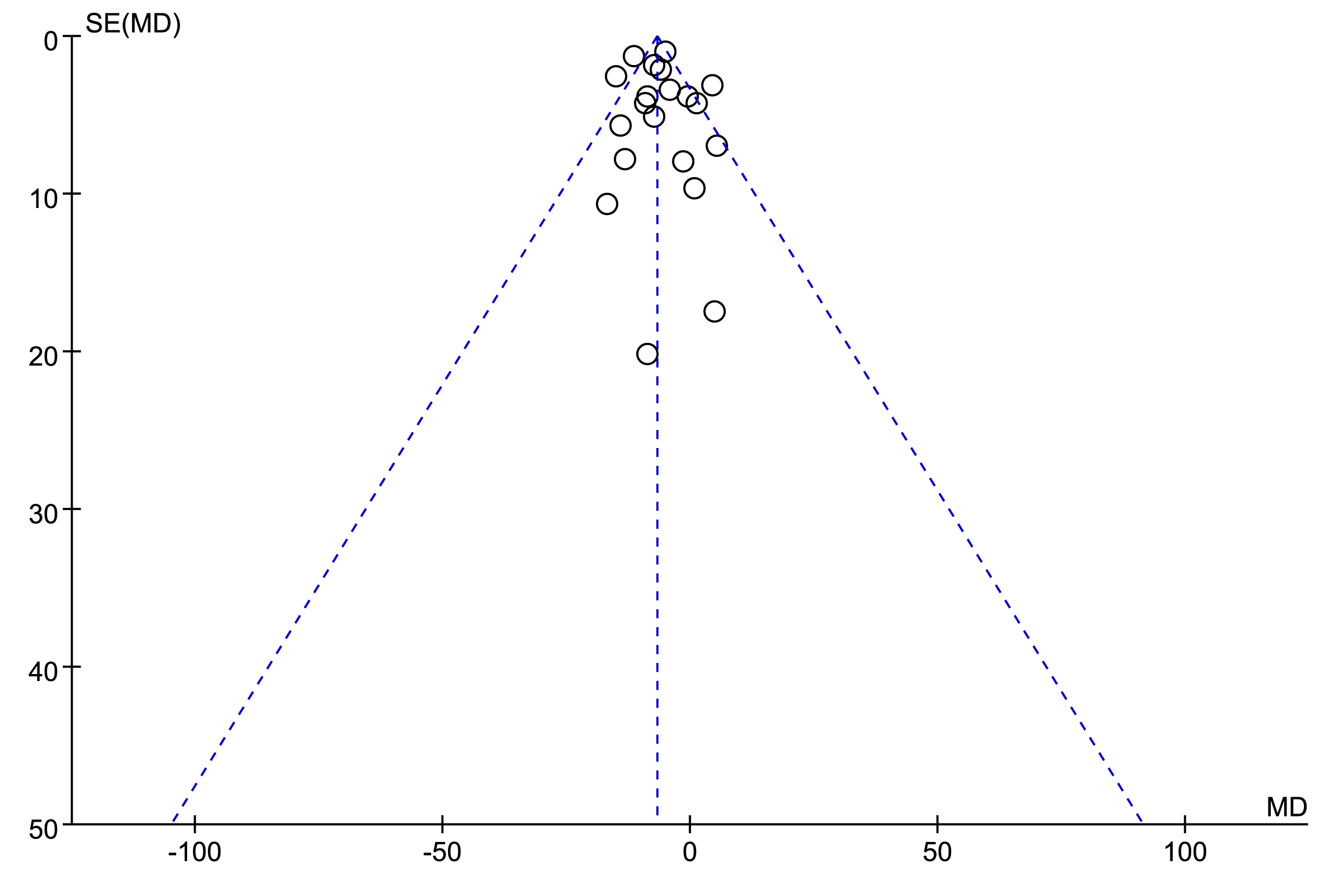


**(A) Grade of hepatic steatosis change.** Egger’s test was adopted and P >0.05.

**(B) ALT (U/L) change.** Egger’s test was adopted and P >0.05.


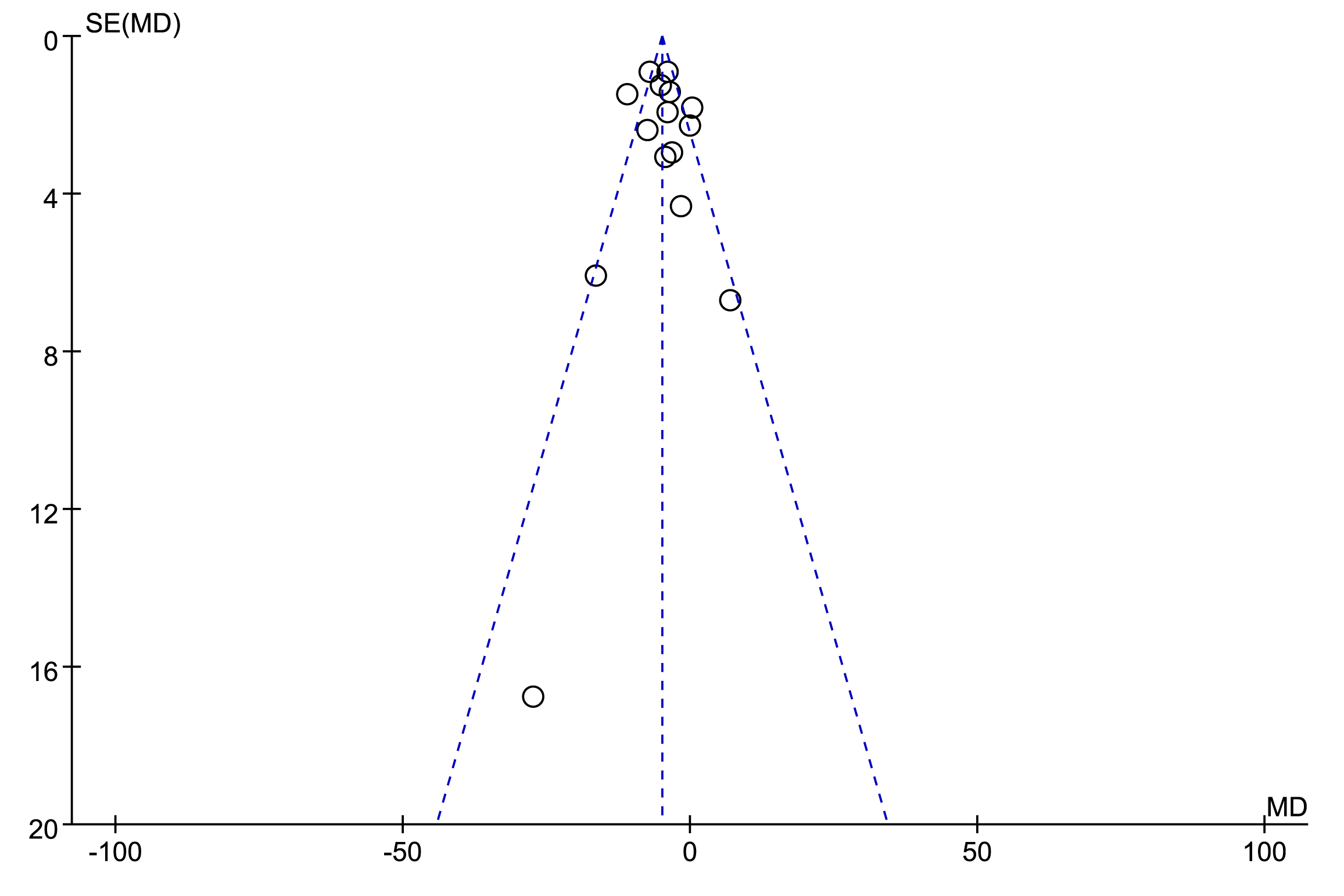

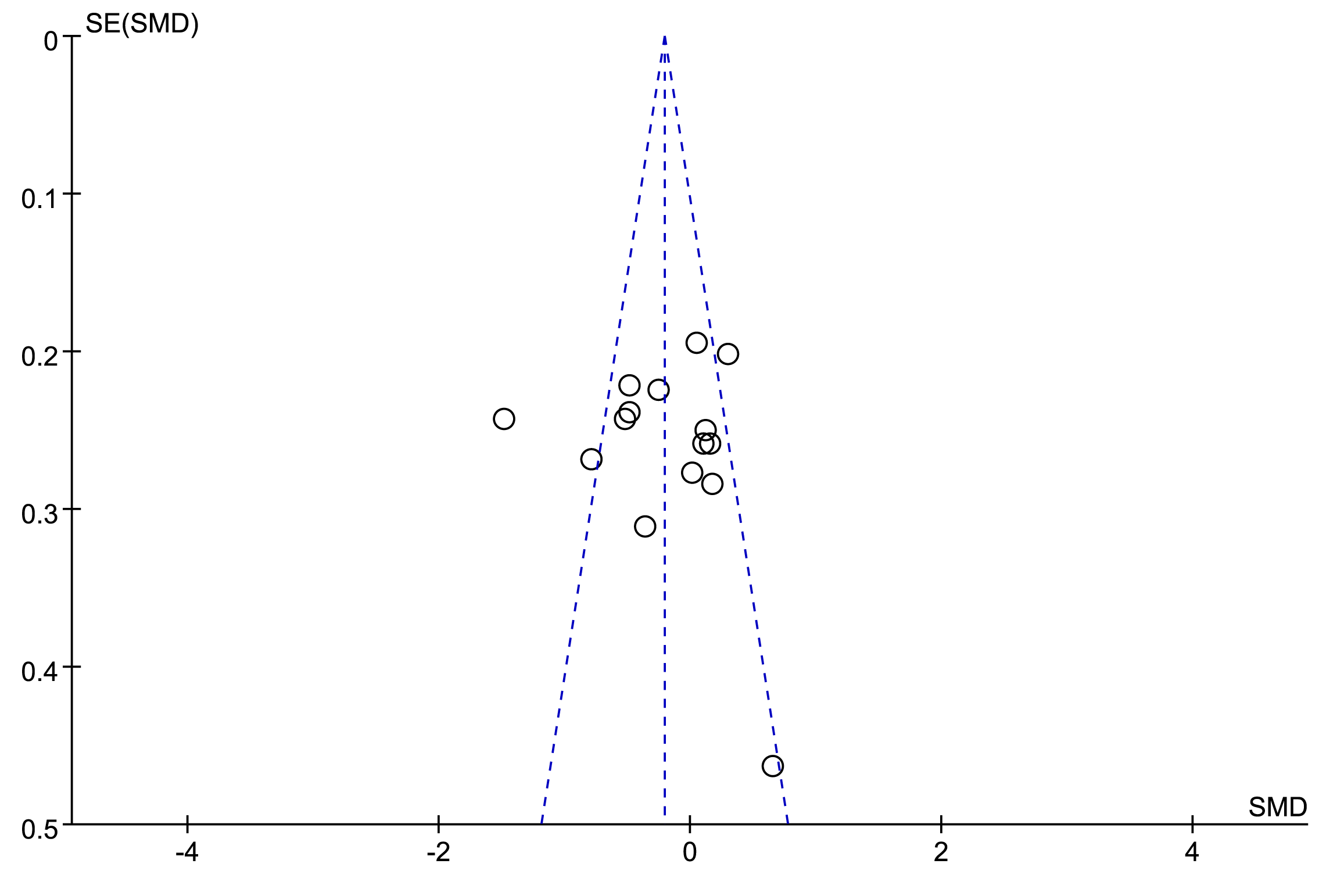


**(D) TC change.** Egger’s test was adopted and P >0.05.

**(C) AST (U/L) change.** Egger’s test was adopted and P >0.05.


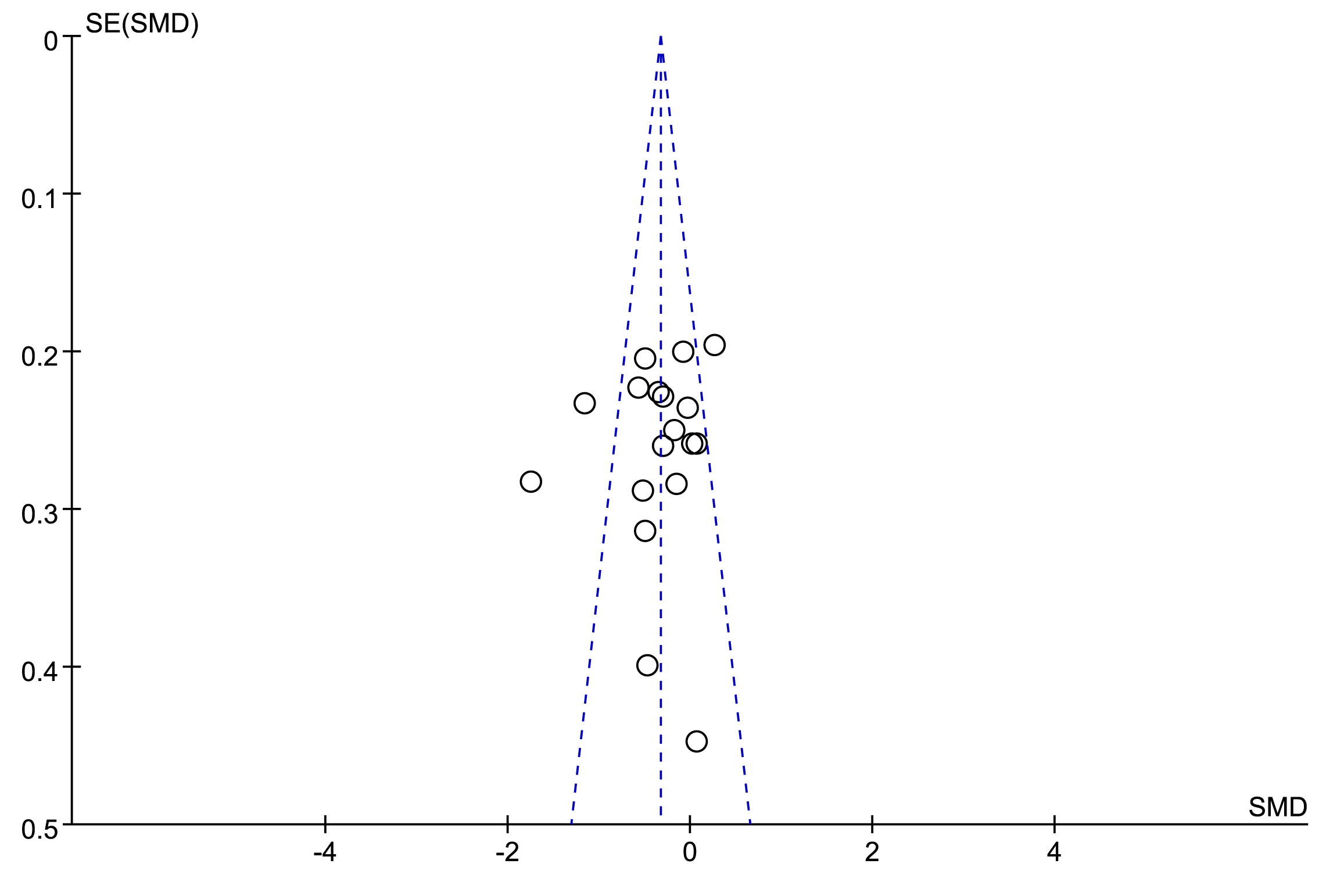

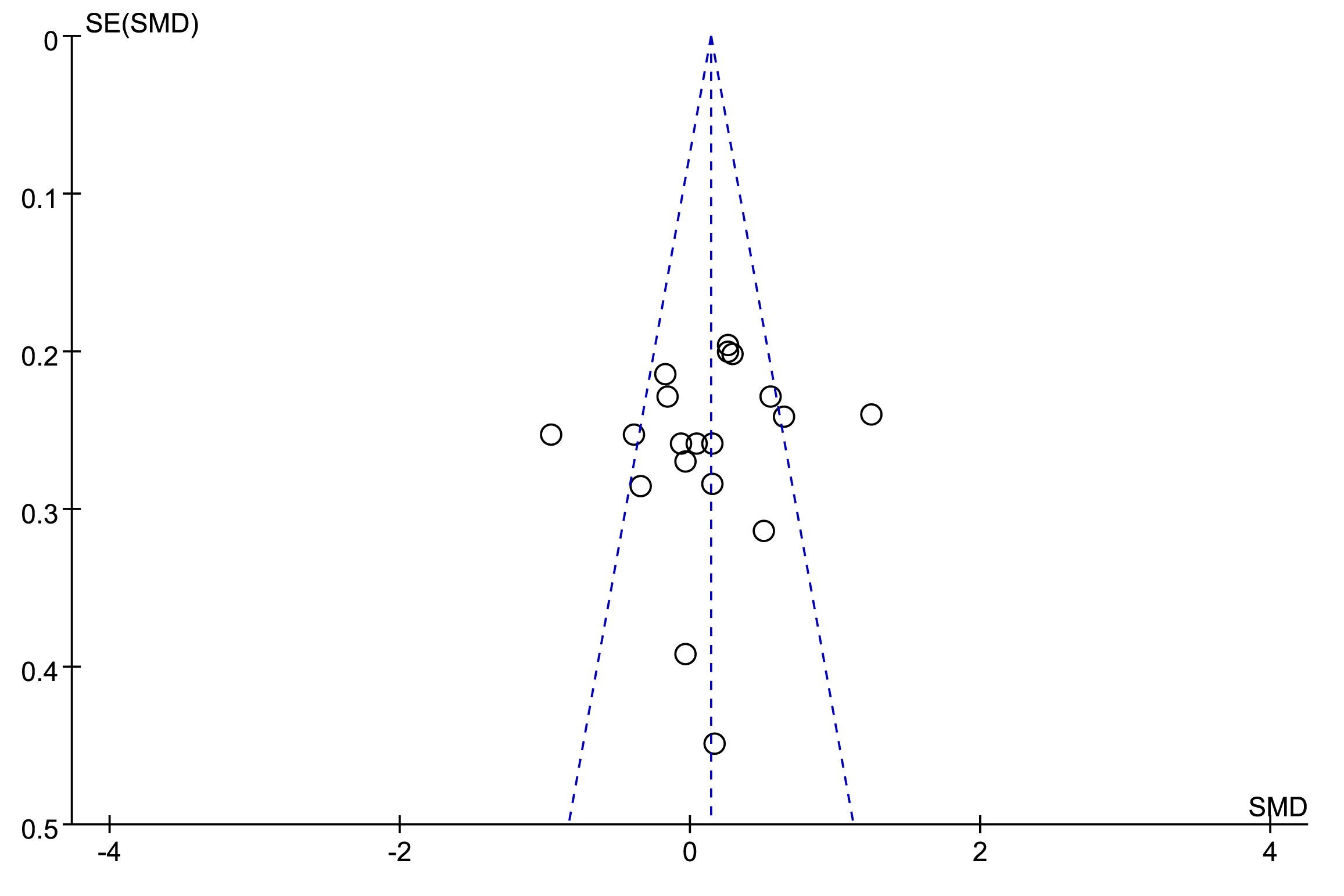


**(F) HDL-C change.** Egger’s test was adopted and P >0.05.

**(E) TG change.** Egger’s test was adopted and P >0.05.


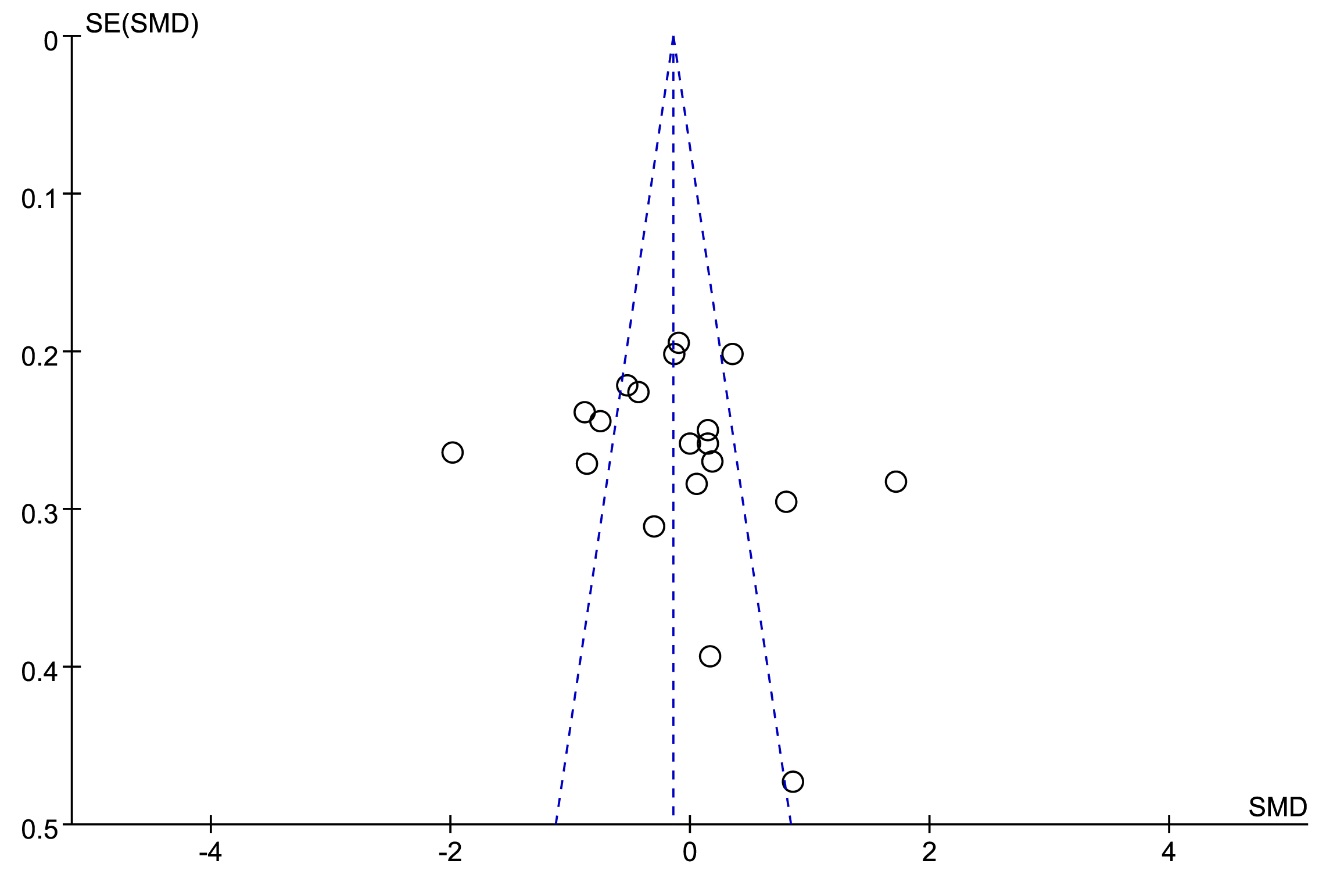

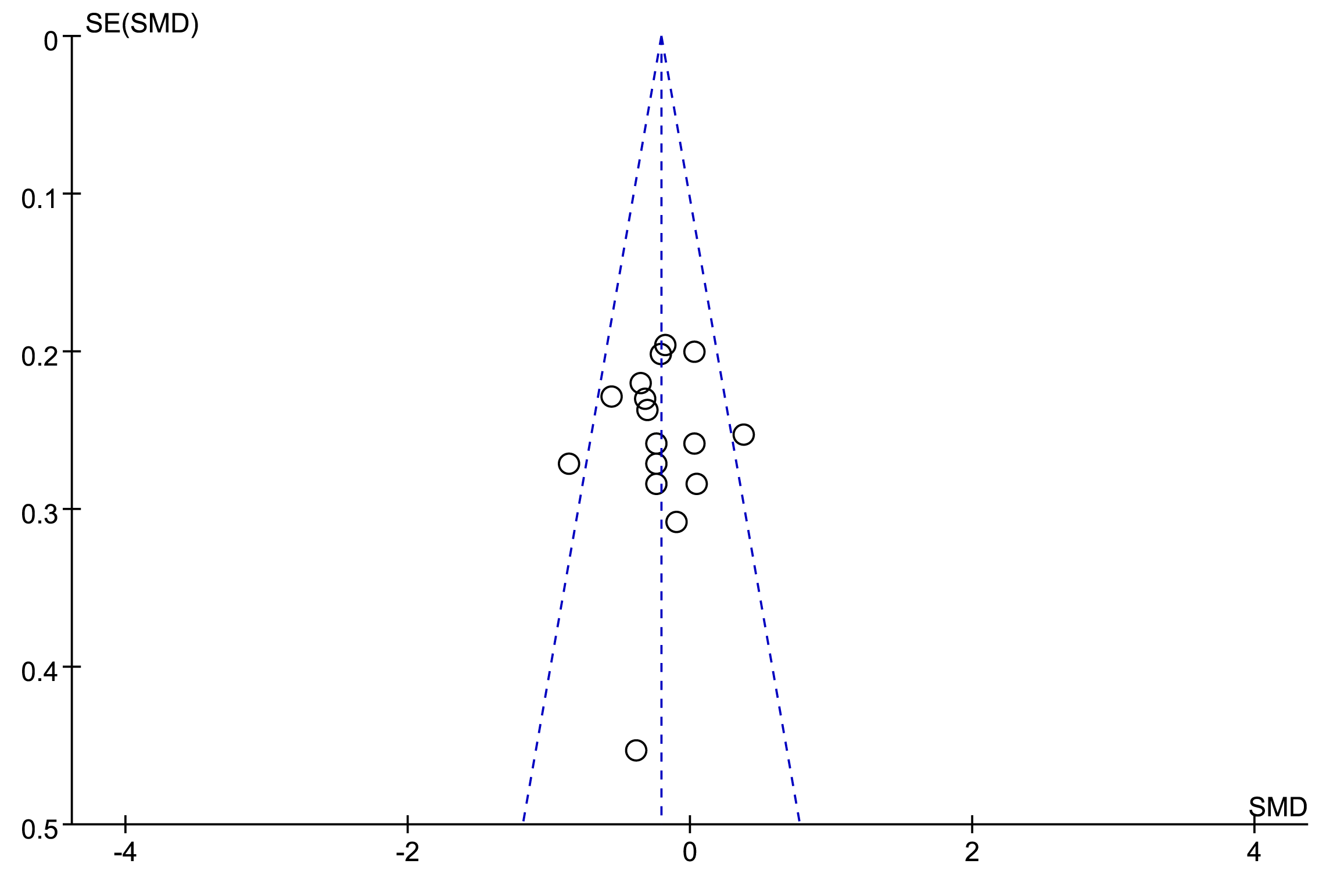


**(G) LDL-C change.** Egger’s test was adopted and P >0.05.

**(H) BMI (kg/m^2^) change.** Egger’s test was adopted and P >0.05.


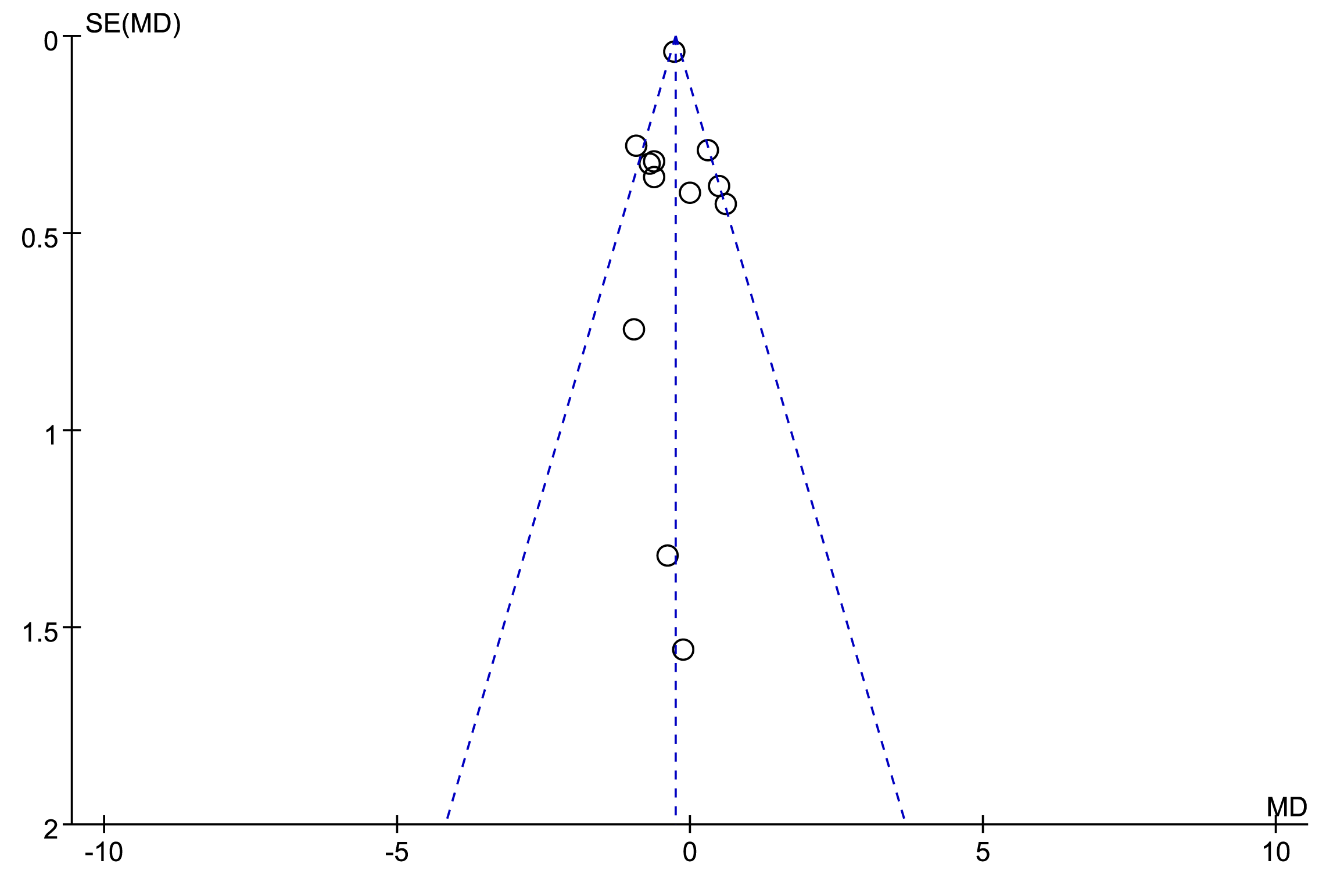

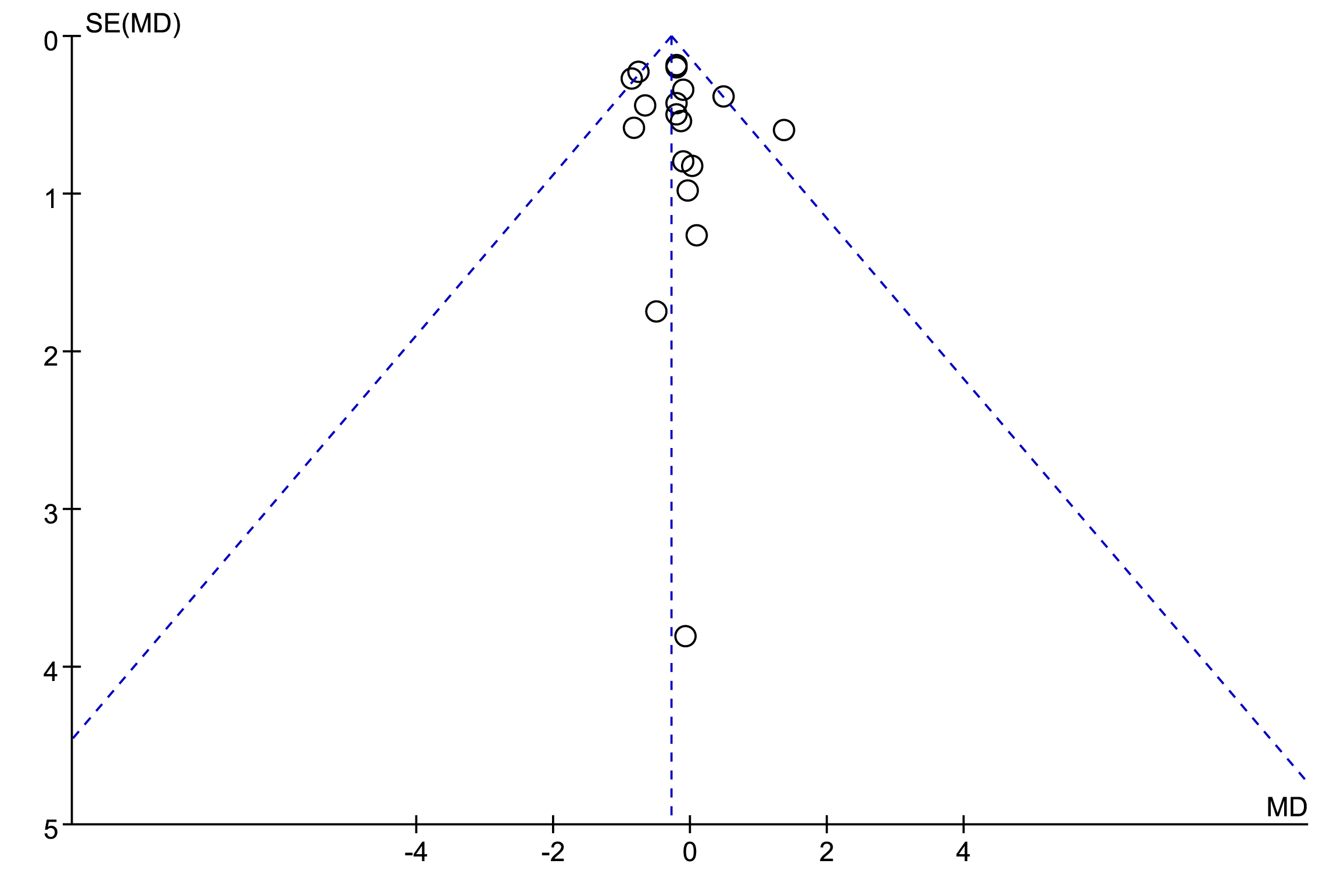


**(I) FBG change.** Egger’s test was adopted and P >0.05.

**(J) HOMA-IR change.** Egger’s test was adopted and P >0.05.


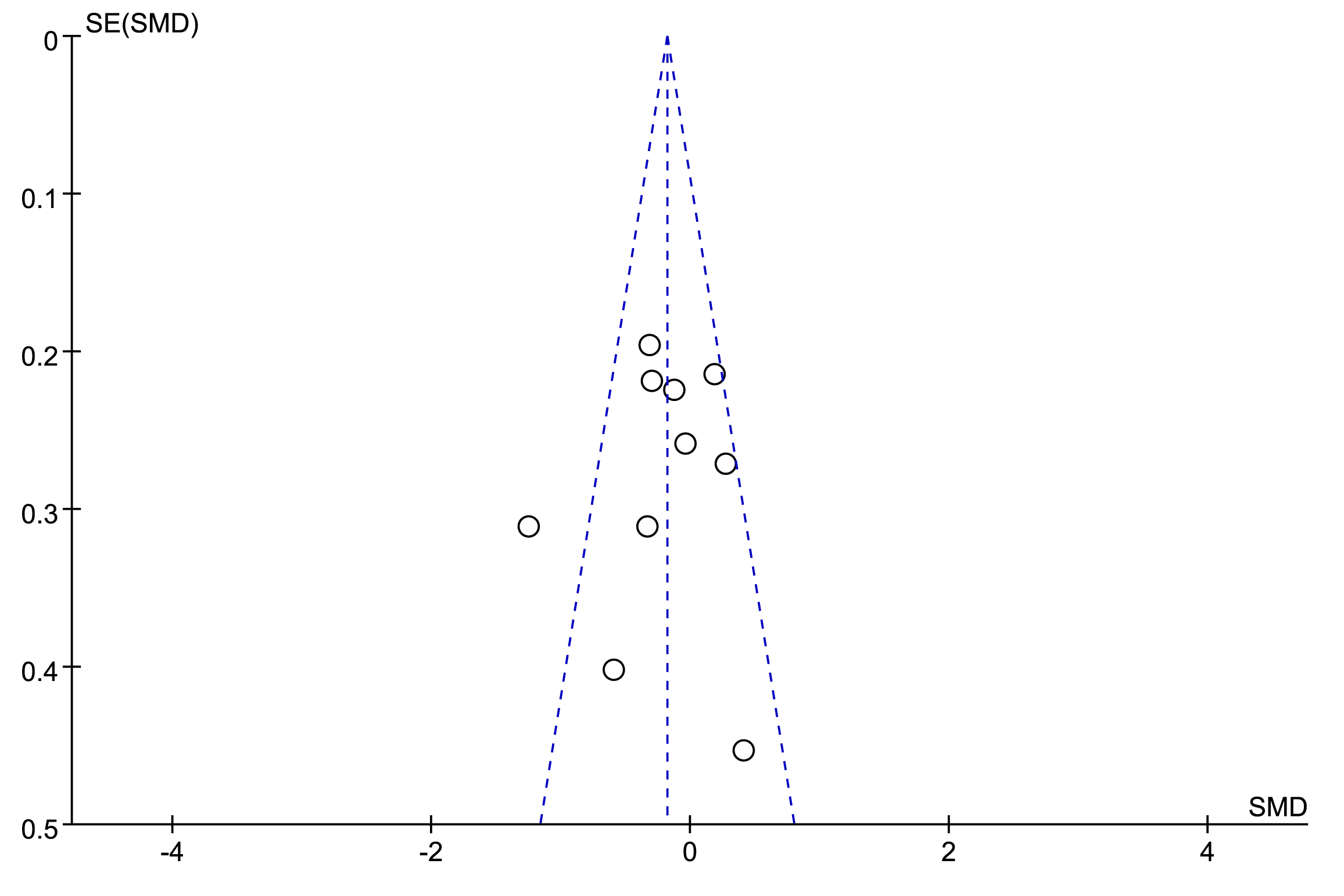

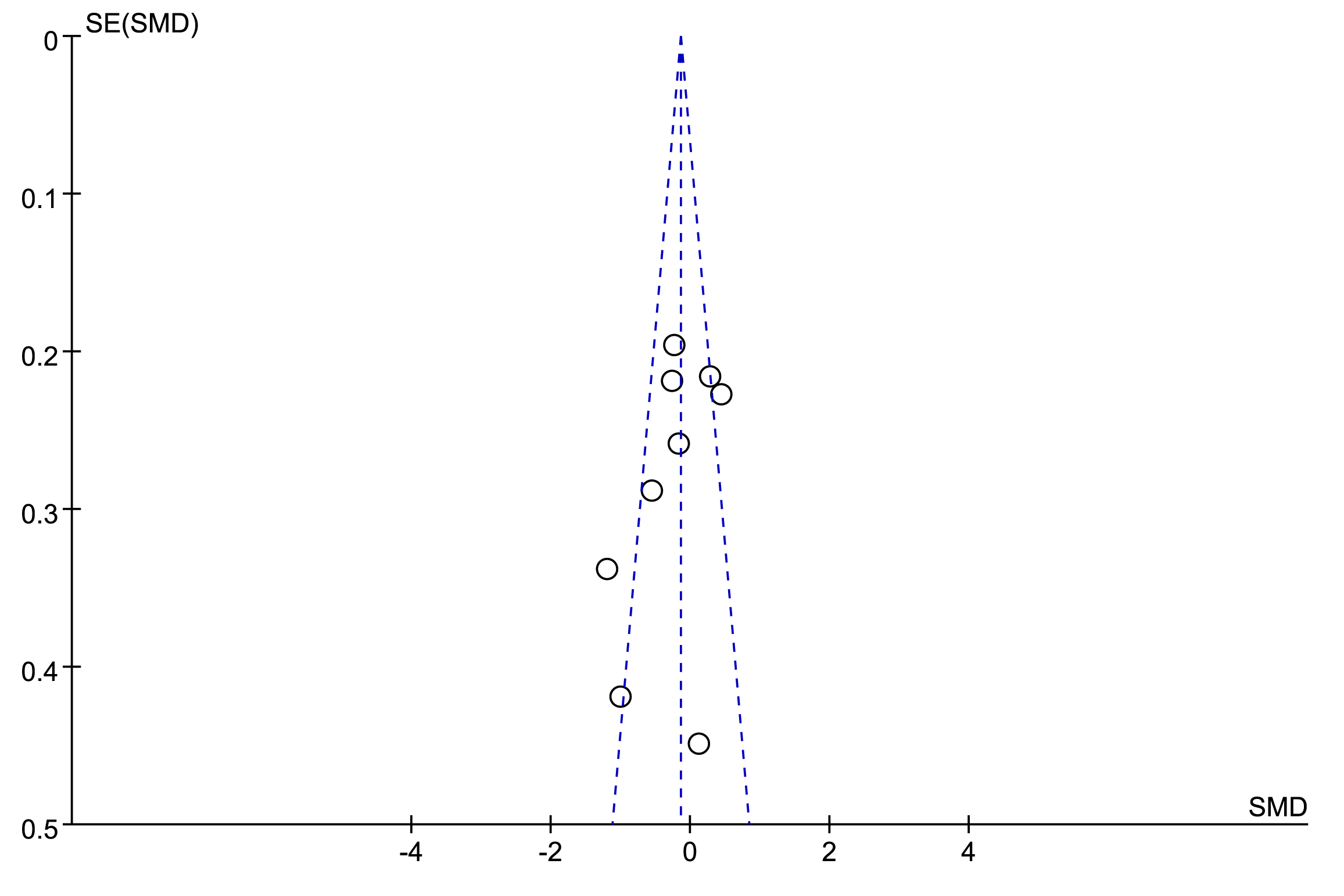


**(K) FBG change.** Egger’s test was adopted and P >0.05.

**(L) FBG change.** Egger’s test was adopted and P >0.05.

Supplement S4: Search terms

**PubMed Advanced search：30 articles**

Search number Query Sort By Filters Search Details Results Time

Search number Query Sort By Filters Search Details Results

6 ((("Non-alcoholic Fatty Liver Disease"[Mesh]) OR (((((((((((((Non alcoholic Fatty Liver Disease[Title/Abstract]) OR (NAFLD[Title/Abstract])) OR (Nonalcoholic Fatty Liver Disease[Title/Abstract])) OR (Fatty Liver, Nonalcoholic[Title/Abstract])) OR (Fatty Livers, Nonalcoholic[Title/Abstract])) OR (Liver, Nonalcoholic Fatty[Title/Abstract])) OR (Livers, Nonalcoholic Fatty[Title/Abstract])) OR (Nonalcoholic Fatty Liver[Title/Abstract])) OR (Nonalcoholic Fatty Livers[Title/Abstract])) OR (Nonalcoholic Steatohepatitis[Title/Abstract])) OR (Nonalcoholic Steatohepatitides[Title/Abstract])) OR (Steatohepatitides, Nonalcoholic[Title/Abstract])) OR (Steatohepatitis, Nonalcoholic[Title/Abstract]))) AND (((((((active ingredient[Title/Abstract]) OR (bioactive ingredients[Title/Abstract])) OR (active compounds[Title/Abstract])) OR (active components[Title/Abstract])) OR (compound[Title/Abstract])) OR (natural compounds[Title/Abstract])) OR (natural products[Title/Abstract]))) AND (randomized controlled trial[Publication Type] OR randomized[Title/Abstract] OR placebo[Title/Abstract]) ("Non-alcoholic Fatty Liver Disease"[MeSH Terms] OR ("Non-alcoholic Fatty Liver Disease"[Title/Abstract] OR "NAFLD"[Title/Abstract] OR "nonalcoholic fatty liver disease"[Title/Abstract] OR "fatty liver nonalcoholic"[Title/Abstract] OR (("fatty liver"[MeSH Terms] OR ("Fatty"[All Fields] AND "Liver"[All Fields]) OR "fatty liver"[All Fields] OR ("Fatty"[All Fields] AND "Livers"[All Fields]) OR "fatty livers"[All Fields]) AND "Nonalcoholic"[Title/Abstract]) OR "liver nonalcoholic fatty"[Title/Abstract] OR (("Liver"[MeSH Terms] OR "Liver"[All Fields] OR "Livers"[All Fields] OR "liver s"[All Fields]) AND "nonalcoholic fatty"[Title/Abstract]) OR "nonalcoholic fatty liver"[Title/Abstract] OR "nonalcoholic fatty livers"[Title/Abstract] OR "nonalcoholic steatohepatitis"[Title/Abstract] OR (("fatty liver"[MeSH Terms] OR ("Fatty"[All Fields] AND "Liver"[All Fields]) OR "fatty liver"[All Fields]) AND "Nonalcoholic"[Title/Abstract]) OR "steatohepatitis nonalcoholic"[Title/Abstract])) AND ("active ingredient"[Title/Abstract] OR "bioactive ingredients"[Title/Abstract] OR "active compounds"[Title/Abstract] OR "active components"[Title/Abstract] OR "compound"[Title/Abstract] OR "natural compounds"[Title/Abstract] OR "natural products"[Title/Abstract]) AND ("randomized controlled trial"[Publication Type] OR "randomized"[Title/Abstract] OR "placebo"[Title/Abstract]) 30

5 randomized controlled trial[Publication Type] OR randomized[Title/Abstract] OR placebo[Title/Abstract] "randomized controlled trial"[Publication Type] OR "randomized"[Title/Abstract] OR "placebo"[Title/Abstract] 950,841

4 ((((((active ingredient[Title/Abstract]) OR (bioactive ingredients[Title/Abstract])) OR (active compounds[Title/Abstract])) OR (active components[Title/Abstract])) OR (compound[Title/Abstract])) OR (natural compounds[Title/Abstract])) OR (natural products[Title/Abstract]) "active ingredient"[Title/Abstract] OR "bioactive ingredients"[Title/Abstract] OR "active compounds"[Title/Abstract] OR "active components"[Title/Abstract] OR "compound"[Title/Abstract] OR "natural compounds"[Title/Abstract] OR "natural products"[Title/Abstract] 433,966

3 ("Non-alcoholic Fatty Liver Disease"[Mesh]) OR (((((((((((((Non alcoholic Fatty Liver Disease[Title/Abstract]) OR (NAFLD[Title/Abstract])) OR (Nonalcoholic Fatty Liver Disease[Title/Abstract])) OR (Fatty Liver, Nonalcoholic[Title/Abstract])) OR (Fatty Livers, Nonalcoholic[Title/Abstract])) OR (Liver, Nonalcoholic Fatty[Title/Abstract])) OR (Livers, Nonalcoholic Fatty[Title/Abstract])) OR (Nonalcoholic Fatty Liver[Title/Abstract])) OR (Nonalcoholic Fatty Livers[Title/Abstract])) OR (Nonalcoholic Steatohepatitis[Title/Abstract])) OR (Nonalcoholic Steatohepatitides[Title/Abstract])) OR (Steatohepatitides, Nonalcoholic[Title/Abstract])) OR (Steatohepatitis, Nonalcoholic[Title/Abstract])) "Non-alcoholic Fatty Liver Disease"[MeSH Terms] OR ("Non-alcoholic Fatty Liver Disease"[Title/Abstract] OR "NAFLD"[Title/Abstract] OR "nonalcoholic fatty liver disease"[Title/Abstract] OR "fatty liver nonalcoholic"[Title/Abstract] OR (("fatty liver"[MeSH Terms] OR ("Fatty"[All Fields] AND "Liver"[All Fields]) OR "fatty liver"[All Fields] OR ("Fatty"[All Fields] AND "Livers"[All Fields]) OR "fatty livers"[All Fields]) AND "Nonalcoholic"[Title/Abstract]) OR "liver nonalcoholic fatty"[Title/Abstract] OR (("Liver"[MeSH Terms] OR "Liver"[All Fields] OR "Livers"[All Fields] OR "liver s"[All Fields]) AND "nonalcoholic fatty"[Title/Abstract]) OR "nonalcoholic fatty liver"[Title/Abstract] OR "nonalcoholic fatty livers"[Title/Abstract] OR "nonalcoholic steatohepatitis"[Title/Abstract] OR (("fatty liver"[MeSH Terms] OR ("Fatty"[All Fields] AND "Liver"[All Fields]) OR "fatty liver"[All Fields]) AND "Nonalcoholic"[Title/Abstract]) OR "steatohepatitis nonalcoholic"[Title/Abstract]) 34,426

2 ((((((((((((Non alcoholic Fatty Liver Disease[Title/Abstract]) OR (NAFLD[Title/Abstract])) OR (Nonalcoholic Fatty Liver Disease[Title/Abstract])) OR (Fatty Liver, Nonalcoholic[Title/Abstract])) OR (Fatty Livers, Nonalcoholic[Title/Abstract])) OR (Liver, Nonalcoholic Fatty[Title/Abstract])) OR (Livers, Nonalcoholic Fatty[Title/Abstract])) OR (Nonalcoholic Fatty Liver[Title/Abstract])) OR (Nonalcoholic Fatty Livers[Title/Abstract])) OR (Nonalcoholic Steatohepatitis[Title/Abstract])) OR (Nonalcoholic Steatohepatitides[Title/Abstract])) OR (Steatohepatitides, Nonalcoholic[Title/Abstract])) OR (Steatohepatitis, Nonalcoholic[Title/Abstract]) "non alcoholic fatty liver disease"[Title/Abstract] OR "NAFLD"[Title/Abstract] OR "nonalcoholic fatty liver disease"[Title/Abstract] OR "fatty liver nonalcoholic"[Title/Abstract] OR (("fatty liver"[MeSH Terms] OR ("Fatty"[All Fields] AND "Liver"[All Fields]) OR "fatty liver"[All Fields] OR ("Fatty"[All Fields] AND "Livers"[All Fields]) OR "fatty livers"[All Fields]) AND "Nonalcoholic"[Title/Abstract]) OR "liver nonalcoholic fatty"[Title/Abstract] OR (("Liver"[MeSH Terms] OR "Liver"[All Fields] OR "Livers"[All Fields] OR "liver s"[All Fields]) AND "nonalcoholic fatty"[Title/Abstract]) OR "nonalcoholic fatty liver"[Title/Abstract] OR "nonalcoholic fatty livers"[Title/Abstract] OR "nonalcoholic steatohepatitis"[Title/Abstract] OR (("fatty liver"[MeSH Terms] OR ("Fatty"[All Fields] AND "Liver"[All Fields]) OR "fatty liver"[All Fields]) AND "Nonalcoholic"[Title/Abstract]) OR "steatohepatitis nonalcoholic"[Title/Abstract] 32,799

1 "Non-alcoholic Fatty Liver Disease"[Mesh] Most Recent "Non-alcoholic Fatty Liver Disease"[MeSH Terms] 19,193

**Cochrane Database of Systematic Reviews：241 articles (including two Meta-articles)**

#1 MeSH descriptor: [Non-alcoholic Fatty Liver Disease] explode all trees 1253

#2 (Steatohepatitis, Nonalcoholic):ti,ab,kw OR (Steatohepatitides, Nonalcoholic):ti,ab,kw OR (Nonalcoholic Steatohepatitis):ti,ab,kw OR (Nonalcoholic Steatohepatitides):ti,ab,kw OR (Fatty Livers, Nonalcoholic):ti,ab,kw OR (Liver, Nonalcoholic Fatty):ti,ab,kw OR (Nonalcoholic Fatty Livers):ti,ab,kw OR (Livers, Nonalcoholic Fatty):ti,ab,kw OR (NAFLD):ti,ab,kw OR (Non alcoholic Fatty Liver Disease):ti,ab,kw OR (Nonalcoholic Fatty Liver):ti,ab,kw OR (Nonalcoholic Fatty Liver Disease):ti,ab,kw OR (Fatty Liver, Nonalcoholic):ti,ab,kw 3876

#3 #1 OR #2 3876

#4 (active ingredient):ti,ab,kw OR (bioactive ingredients):ti,ab,kw OR (active compounds):ti,ab,kw OR (active components):ti,ab,kw OR (compound):ti,ab,kw OR (natural compounds):ti,ab,kw OR (natural products):ti,ab,kw 57494

#5 (randomized controlled trial):ti,ab,kw OR (randomized):ti,ab,kw OR (placebo):ti,ab,kw 1104534

#6 #3 AND #4 AND #5 241

**Embase：50 articles**

#6 #3 AND #4 AND #5 50

#5 'randomized controlled trial':ab,ti OR 'randomized':ab,ti OR 'placebo':ab,ti 1043510 21-May-22

#4 active ingredient':ab,ti OR 'bioactive ingredients':ab,ti OR 'active compounds':ab,ti OR 'active components':ab,ti OR 'compound':ab,ti OR 'natural compounds':ab,ti OR 'natural products':ab,ti 497089 21-May-22

#3 #1 OR #2 63347 21-May-22

#2 nafld':ab,ti OR 'non alcoholic fatty liver disease':ab,ti OR 'non alcoholic hepato-steatosis':ab,ti OR 'non alcoholic hepatosteatosis':ab,ti OR 'non alcoholic liver steatosis':ab,ti OR 'non alcoholic steatotic hepatopathy':ab,ti OR 'non-alcoholic fatty liver':ab,ti OR 'non-alcoholic fatty liver disease':ab,ti OR 'non-alcoholic fld':ab,ti OR 'non-alcoholic hepatic steatosis':ab,ti OR 'nonalcoholic fatty liver disease':ab,ti OR 'nonalcoholic fld':ab,ti OR 'nonalcoholic hepatic steatosis':ab,ti OR 'nonalcoholic hepatosteatosis':ab,ti OR 'nonalcoholic liver steatosis':ab,ti 44136 21-May-22

#1 nonalcoholic fatty liver'/exp 57209 21-May-22

**Web of Science：159 articles**

(nonalcoholic fatty liver OR nafld OR non alcoholic fatty liver disease OR non alcoholic hepato-steatosis OR non alcoholic hepatosteatosis OR non alcoholic liver steatosis OR non alcoholic steatotic hepatopathy OR non-alcoholic fatty liver OR non-alcoholic fatty liver disease OR non-alcoholic fld OR non-alcoholic hepatic steatosi OR nonalcoholic fatty liver disease OR nonalcoholic fld OR nonalcoholic hepatic steatosi OR nonalcoholic hepatosteatosis OR nonalcoholic liver steatosis) AND (active ingredient OR 'bioactive ingredients OR active compounds OR 'active components OR compound OR natural compounds OR 'natural products) AND (randomized controlled trial OR 'randomized OR placebo)

**China national knowledge internet (CNKI)：78articles**

Search strategy： ( ( (主题=脂肪肝 或者 题名=脂肪肝 或者 v_subject=中英文扩展(脂肪肝) 或者 title=中英文扩展(脂肪肝)) 或者 (主题=脂肪性肝炎 或者 题名=脂肪性肝炎 或者 v_subject=中英文扩展(脂肪性肝炎) 或者 title=中英文扩展(脂肪性肝炎)) ) 或者 ( (主题=脂肪性肝病 或者 题名=脂肪性肝病 或者 v_subject=中英文扩展(脂肪性肝病) 或者 title=中英文扩展(脂肪性肝病)) 或者 (主题=中英文扩展(NAFLD) 或者 题名=中英文扩展(NAFLD) 或者 v_subject=NAFLD 或者 title=NAFLD) ) ) 并且 ( ( (主题=单体 或者 题名=单体 或者 v_subject=中英文扩展(单体) 或者 title=中英文扩展(单体)) 或者 (主题=活性成分 或者 题名=活性成分 或者 v_subject=中英文扩展(活性成分) 或者 title=中英文扩展(活性成分)) ) 或者 ( (主题=化合物 或者 题名=化合物 或者 v_subject=中英文扩展(化合物) 或者 title=中英文扩展(化合物)) 或者 (主题=有效成分 或者 题名=有效成分 或者 v_subject=中英文扩展(有效成分) 或者 title=中英文扩展(有效成分)) ) ) 并且 ( (摘要=中英文扩展(RCT) 或者 abstract_en=RCT) 或者 (摘要=随机 或者 abstract_en=中英文扩展(随机)) ) (模糊匹配)

**Chinese Biomedical Literature Database (CBM) search strategy: 177 articles**

Search strategy：(("随机"[常用字段:智能]) OR ("随机对照试验"[不加权:扩展])) AND (("有效成分"[常用字段:智能]) OR ("化合物"[常用字段:智能]) OR ("单体"[常用字段:智能]) OR ("活性成分"[常用字段:智能])) AND (("NAFLD"[常用字段:智能]) OR ("脂肪性肝炎"[常用字段:智能]) OR ("脂肪性肝病"[常用字段:智能]) OR ("脂肪肝"[不加权:扩展]))
